# Quantized Plasmon Modes for Metallic Nanoparticles of Arbitrary Shape with a Generic Dielectric Function

**DOI:** 10.1021/acs.jpclett.6c02006

**Published:** 2026-08-28

**Authors:** Marco Romanelli, Gabriel Gil, Stefano Corni

**Affiliations:** † Department of Chemical Sciences, 9308 University of Padova, via Marzolo 1, 35131 Padova, Italy; ‡ Dipartimento di Scienze e Innovazione Tecnologica, 201330 Università degli Studi del Piemonte Orientale Amedeo Avogadro, V.le Teresa Michel 11, 15121 Alessandria, Italy; ¶ CNR Institute of Nanoscience, via Campi 213/A, 41125 Modena, Italy; § Padua Quantum Technologies Research Center, 9308 University of Padova, 35131 Padova, Italy

## Abstract

In this work, we introduce an effective approach to quantize the electromagnetic response of plasmonic metallic nanostructures. Their shape is arbitrary, and they feature a realistic description of the frequency-dependent metal dielectric function that is based on experimental data. The derived quantum modes correctly reproduce the linear response macroscopic polarization of the nanoparticle upon external drive according to classical Maxwell’s equations in the quasistatic limit. We further investigated the coupling of these modes to a quantum-chemical molecular description. The presented methodology paves the way for accurate modeling of a plexcitonic system, where strong plasmon–molecule coupling and/or strong-driving fields call for a quantized description of the plasmonic response.

## Graphical abstract

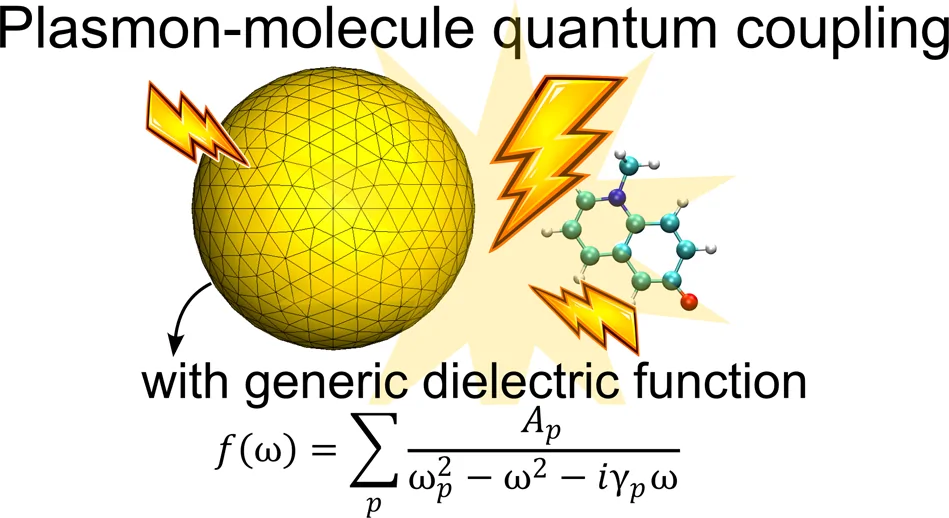

Plasmonic metallic nanoparticles (NPs) exhibit coherent and collective oscillations of the metal conduction electrons upon light irradiation. Such phenomenon, referred to as Localized Surface Plasmon Resonance (LSPR), leads to the enhancement of the external electromagnetic field in the proximity of the metal surface, down to molecular scale.^1–3^ Over the past decades there has been rising interest in making use of NPs to shape molecular properties by coupling electronic transitions of molecules with the plasmon-enhanced local electromagnetic field arising from LSPRs. This area, known as molecular plasmonics,^4^ has proved to be an effective noninvasive way of modulating molecular properties as a result of light–matter coupling at the nanoscale. Different applications have been illustrated, showcasing the capability of plasmonic systems to sizably modify molecular photoluminescence,^5–9^ Raman scattering,^10–12^ molecular energy transfer^13–16^ and excited-state decay,^17–19^ as well as to promote few-emitter lasing,^20,21^ just to name a few.

In many cases, due to the size difference between molecules and metallic NPs, several state-of-the-art methods^22–24^ tackle these systems by means of multiscale approaches, where the NP response is described by classical electrodynamics while molecules are modeled at the ab initio level. Fully first-principles approaches, as well as hybrid methods combining atomistic and electrodynamic descriptions, provide valuable insights into the electronic structure and plasmonic properties of nanoparticles, but their application is often limited to smaller systems due to the rapidly increasing computational cost.^25,26^ Therefore, classical electrodynamics NP models become particularly valuable when realistically large NPs are considered. Such a classical description of the NP is physically appropriate in the weak plasmon–molecule coupling regime and, depending on the target observables, often also in the single-excitation manifold under strong coupling.^27^ Nevertheless, whenever an explicit treatment of the system excited states is needed, for instance to investigate the effect of nuclear motion on the dynamics of a polaritonic or plexcitonic system,^23^ an accurate evaluation of the light–matter coupled potential energy surfaces becomes necessary, which in turn entails a quantum description of the plasmonic response. Additionally, spontaneous emission processes, such as few-emitter lasing,^20,21^ also require a quantum treatment of the plasmonic response. Furthermore, under high-intensity driving fields, accurately describing the evolution of the molecular excited-state populations within a classical framework is generally not straightforward.^28,29^ Under these conditions, a quantum description of the NP response is necessary to capture the correct system dynamics.

In the following work, building on a boundary element method (BEM) approach to solve classical macroscopic Maxwell equations in the quasistatic limit, we introduce effective quantum modes to model the optical response of arbitrarily shaped NPs described by a generic dielectric function.

Similar approaches based on continuum solutions of the electrodynamics problem were previously introduced^30–33^ and later successfully coupled to a quantum chemistry molecular description.^34,35^ However, in the latter case the metal dielectric function is typically described by a simple Drude or Drude–Lorentz (DL) form, thereby constituting a rough approximation to real metal dielectric functions. Indeed, it is well-known that for widely used plasmonic metals, like silver or gold, multiple interband transitions fall in the same spectral region of free-electrons plasmonic resonances, thus making those analytical models inaccurate to properly describe the real dielectric response. To overcome this limitation, the generic dielectric function approach, which relies on fitting a sum of DL oscillators to experimental values of frequency-dependent metal dielectric functions, was recently introduced in a BEM framework,^36^ opening up the possibility to investigate the electron dynamics of molecules in realistic plasmonic environments. To date, the application of this generic dielectric function approach has been restricted to the classical BEM problem. The main objective of the present work is to develop a general quantization of this formalism, enabling a quantum description of plasmonic systems with arbitrary dielectric responses within the BEM framework. The illustrative application presented below is intended to highlight the generality and flexibility of the proposed approach, as well as its capabilities with respect to previous formulations,^35^ rather than to emphasize regimes in which the quantum description departs from the classical one.^28,29^

While macroscopic quantum electrodynamics (QED) provides a way to quantize the electromagnetic field (EM) in such environments by introducing a continuum of harmonic oscillators,^37^ its practical application is limited to cases where perturbative approximations are valid. Indeed, recent works^38–40^ have shown that such complex EM continuum structure can be effectively represented by a set of discrete modes that are lossy and coupled and stem from a system-specific fitting procedure based on the spectral density of the photonic/plasmonic environment of interest. We hereby show that such few-mode effective quantization of the EM continuum via discrete, coupled and lossy modes naturally arises in a quasistatic BEM approach in a way that depends on the dielectric response of the bulk metal, with the effect of the NP shape accounted for numerically but without the need for a fitting procedure. This leads to the identification of the linear response matrix of a quantum system from which quantization of the NP response is achieved. This result shows that the possibility of writing the response of a NP in terms of a set of discrete, coupled and lossy modes is a general feature of nanostructures, at least in the quasi-static response framework.

In the quasistatic BEM approach that we consider, also termed Polarizable Continuum Model - Nanoparticle (PCM-NP),^22^ the linear response polarization of a given NP due to an external electric field is expressed in terms of a surface charge density lying on the NP surface. The problem of finding such surface charge density for a given driving field is solved numerically using a BEM strategy based on a tessellation (discretization) of the NP surface. The BEM response equation reads

q(ω)=Q(ω)V(ω) 1

where ***q*** (ω) and ***V*** (ω) are vectors collecting the polarization charges and the electrostatic potential associated with the external electric field acting at each surface element, respectively. On the other hand, ***Q*** (ω) is the PCM-NP response matrix defined as

$$Q\left(\omega \right)=-S^{-1}{\left(2\pi \frac{\epsilon \left(\omega \right)+1}{\epsilon \left(\omega \right)-1}+\mathrm{DA}\right)}^{-1}\left(2\pi I+\mathrm{DA}\right)$$ 2

where ***S*** and ***D*** are the Calderon matrices with elements^41^

$$S_{ij}=\frac{1}{\left|{\mathrm{s⃗}}_{i}-{\mathrm{s⃗}}_{j}\right|}$$ 3

$$S_{ii}=1.0694\sqrt{A_{ii}/4\pi }$$ 4

$$D_{ij}=\frac{\left({\mathrm{s⃗}}_{i}-{\mathrm{s⃗}}_{j}\right)\cdot {\mathrm{n⃗}}_{j}}{|{\mathrm{s⃗}}_{i}-{\mathrm{s⃗}}_{j}{|}^{3}}$$ 5

$$D_{ii}=-\left(2\pi +\underset{k\neq i}{\sum }D_{ik}A_{kk}\right)/A_{ii}$$ 6

The ***A*** diagonal matrix stores the area of each surface element (also called “tessera”) of the NP discretized surface, whereas ϵ­(ω) is the frequency-dependent dielectric function defining the optical dielectric response.

Following ref 36, eqs 1 and 2 are recast as

$$q\left(\omega \right)=-\frac{1}{2\pi }f\left(\omega \right)\left[AD^{†}q\left(\omega \right)+S^{-1}\left(2\pi I+\mathrm{DA}\right)V\left(\omega \right)\right]$$ 7

with

$$f\left(\omega \right)=\frac{\epsilon \left(\omega \right)-1}{\epsilon \left(\omega \right)+1}$$ 8

For a specific metal, the experimental *f*(ω) data is fitted to a sum of N DL-like poles,

$$f\left(\omega \right)=\frac{\epsilon \left(\omega \right)-1}{\epsilon \left(\omega \right)+1}\approx \overset{N}{\underset{p}{\sum }}\frac{A_{p}}{{\omega_{p}}^{2}-\omega^{2}-i\gamma_{p}\omega }$$ 9

By introducing the fitting functional form of *f*(ω) into eq 7, the polarization charges can be decomposed as pole-dependent charges, i.e., $q\left(\omega \right)=\overset{N}{\underset{p}{\sum }}q_{p}\left(\omega \right)$ , leading to a matrix equation for each pole-dependent charge vector ***q***_*p*_(ω):

$$\frac{2\pi }{A_{p}}\left({\omega_{p}}^{2}-\omega^{2}-i\gamma_{p}\omega \right)q_{p}\left(\omega \right)=-\left[AD^{†}\overset{N}{\underset{p^{\prime }}{\sum }}q_{p\prime }\left(\omega \right)+S^{-1}\left(2\pi I+\mathrm{DA}\right)V\left(\omega \right)\right]$$ 10

As such, the PCM-NP linear response equation within the generic dielectric function approach consists of a problem of coupled damped and driven classical harmonic oscillators. Our goal is therefore to map eq 10 to the linear charge density response of a quantum NP, so that its linear response polarization matches the macroscopic classical one. As shown previously in the simple DL dielectric function case,^35^ such a quantization approach corresponds to a macroscopic-QED quantization of the electromagnetic fields in the same dielectric environment.^30,34,35^

To this end it is convenient to recast eq 9 as

$$f\left(\omega \right)\approx \overset{N}{\underset{p}{\sum }}\frac{A_{p}}{{\omega_{p}}^{2}-\omega^{2}-i\gamma_{p}\omega }=\overset{N}{\underset{p}{\sum }}\frac{A_{p}}{2{\mathrm{\omega ̅}}_{p}}\left(\frac{1}{{\mathrm{\omega ̅}}_{p}-\omega -i\gamma_{p}/2}+\frac{1}{{\mathrm{\omega ̅}}_{p}+\omega +i\gamma_{p}/2}\right)$$ 11

where ${\mathrm{\omega ̅}}_{p}=\sqrt{{\omega_{p}}^{2}-{\gamma_{p}}^{2}/4}$ . Equation 11 allows us to explicitly deal with the resonant and antiresonant terms hidden in eq 9. This is a critical step for obtaining a set of coupled quantum oscillators representing the exact (classical) polarization response of the macroscopic system. Indeed, on the basis of eq 11, the response charges can be decomposed as ***q*** (ω) = ∑_*p*_^*N*^ ***(q***_*p*_^*R*^(ω) + ***q***_*p*_^*A*^(ω)), where ***q***_*p*_^*R*^(ω) and ***q***_*p*_^*A*^(ω), respectively, arise from the resonant and antiresonant terms of eq 11. Similarly to the DL case,^35^ we now express the PCM-NP kernel in its diagonal form via the eigenmode expansion (***S*** ^–1/2^ ***DAS*** ^1/2^ = ***T*** **λ** ***T*** ^†^, note that only the geometry of the NP, not the nature of the material is involved in this step). To clarify the meaning of such eigenmodes, it is useful to note that for a sphere they correspond to surface charge distributions with different multipolar characters (dipole, quadrupole, etc.). Upon using such eigenmode expansion and the decomposition of eq 11, eq 10 can be recast as (SI 1),

$$\left(S^{1/2}TK_{pp}^{R/A}\left(\omega \right)T^{†}S^{1/2}\right)q_{p}^{R/A}\left(\omega \right)+\overset{N}{\underset{p^{\prime }\neq p}{\sum }}\left(S^{1/2}T{\mathrm{\lambda ̃}}_{pp\prime }T^{†}S^{1/2}\right)q_{p\prime }^{R/A}\left(\omega \right)+\overset{N}{\underset{p^{\prime }}{\sum }}\left(S^{1/2}T{\mathrm{\lambda ̃}}_{pp\prime }T^{†}S^{1/2}\right)q_{p\prime }^{A/R}\left(\omega \right)=-V\left(\omega \right)$$ 12

where ***K***_*pp*_^*R*^(ω), ***K***_*pp*_^*A*^(ω) and ***λ̃***_*pp′*_ are diagonal matrices on the surface element eigenmode index θ:

$${\left(K_{pp}^{R}\left(\omega \right)\right)}_{\theta \theta }=\frac{\frac{4\pi {\mathrm{\omega ̅}}_{p}}{A_{p}}\left({\mathrm{\omega ̅}}_{p}-\omega -i\frac{\gamma_{p}}{2}\right)+\lambda_{\theta }}{2\pi +\lambda_{\theta }}$$ 13

$${\left(K_{pp}^{A}\left(\omega \right)\right)}_{\theta \theta }=\frac{\frac{4\pi {\mathrm{\omega ̅}}_{p}}{A_{p}}\left({\mathrm{\omega ̅}}_{p}+\omega +i\frac{\gamma_{p}}{2}\right)+\lambda_{\theta }}{2\pi +\lambda_{\theta }}$$ 14

$${\left({\mathrm{\lambda ̃}}_{pp\prime }\right)}_{\theta \theta }=\frac{\lambda_{\theta }}{2\pi +\lambda_{\theta }}$$ 15

Further manipulation of eqs 12–15 (see SI 1), leads to independent PCM-NP response equations for each θth BEM eigenmode, reading

$$\left[\left(\begin{array}{ll} A_{\theta } & B_{\theta }\\ B_{\theta }^{*} & A_{\theta }^{*} \end{array}\right)-\omega \left(\begin{array}{ll} I & 0\\ 0 & -I \end{array}\right)\right]\left(\begin{array}{l} q_{\theta }^{R}\left(\omega \right)\\ q_{\theta }^{A}\left(-\omega \right) \end{array}\right)=-\left(\begin{array}{l} V_{\theta }\left(\omega \right)\\ V_{\theta }^{*}\left(-\omega \right) \end{array}\right)$$ 16

where $A_{\theta }$ and $B_{\theta }$ are *N* × *N* matrices, whereas $q_{\theta }^{R/A}$ and $V_{\theta }$ are *N*-dimensional vectors whose elements read

$${\left(A_{\theta }\right)}_{pp\prime }=\left({\mathrm{\omega ̅}}_{p}-i\frac{\gamma_{p}}{2}+\lambda_{\theta }\frac{A_{p}}{4\pi {\mathrm{\omega ̅}}_{p}}\right)\delta_{pp\prime }+\left(1-\delta_{pp\prime }\right)\times \sqrt{\frac{A_{p}}{2{\mathrm{\omega ̅}}_{p}}}\frac{\lambda_{\theta }}{2\pi }\sqrt{\frac{A_{p\prime }}{2{\mathrm{\omega ̅}}_{p\prime }}}$$ 17

$${\left(B_{\theta }\right)}_{pp\prime }=\sqrt{\frac{A_{p}}{2{\mathrm{\omega ̅}}_{p}}}\frac{\lambda_{\theta }}{2\pi }{\left(\sqrt{\frac{A_{p\prime \prime }}{2{\mathrm{\omega ̅}}_{p\prime }}}\right)}^{*}$$ 18

$$q_{\theta ,p}^{R}\left(\omega \right)=\frac{1}{\sqrt{\frac{A_{p}}{2{\mathrm{\omega ̅}}_{p}}\left(1+\frac{\lambda_{\theta }}{2\pi }\right)}}\underset{k}{\sum }{\left(T^{†}S^{1/2}\right)}_{\theta k}q_{p,k}^{R}\left(\omega \right)$$ 19

$$q_{\theta ,p}^{A}\left(-\omega \right)=\frac{1}{{\left(\sqrt{\frac{A_{p}}{2{\mathrm{\omega ̅}}_{p}}\left(1+\frac{\lambda_{\theta }}{2\pi }\right)}\right)}^{*}}\underset{k}{\sum }{\left(T^{†}S^{1/2}\right)}_{\theta k}q_{p,k}^{A}\left(\omega \right)$$ 20

$$V_{\theta ,p}\left(\omega \right)=\mathrm{sgn}\left(A_{p}\right){\left(\sqrt{\frac{A_{p}}{2{\mathrm{\omega ̅}}_{p}}\left(1+\frac{\lambda_{\theta }}{2\pi }\right)}\right)}^{*}\times \underset{k}{\sum }{\left(T^{†}S^{-1/2}\right)}_{\theta k}V_{k}\left(\omega \right)$$ 21

$$V_{\theta ,p}^{*}\left(-\omega \right)=\mathrm{sgn}\left(A_{p}\right)\sqrt{\frac{A_{p}}{2{\mathrm{\omega ̅}}_{p}}\left(1+\frac{\lambda_{\theta }}{2\pi }\right)}\times \underset{k}{\sum }{\left(T^{†}S^{-1/2}\right)}_{\theta k}V_{k}\left(\omega \right)$$ 22

Further details on the matrices $A_{\theta }$ and $B_{\theta }$ as well as a graphical representation of their structure is given in SI 1.

Notably, the shape of the classical PCM-NP response equations for each θth BEM eigenmode (eq 16) strongly resembles the shape of the linear response equation of a quantum system (as in time-dependent density functional theory, TDDFT, or time-dependent Hartree–Fock, TDHF),^42–45^

$$\left[\left(\begin{array}{ll} A & B\\ B^{*} & A^{*} \end{array}\right)-\omega \left(\begin{array}{ll} I & 0\\ 0 & -I \end{array}\right)\right]\left(\begin{array}{l} X\left(\omega \right)\\ Y\left(-\omega \right) \end{array}\right)=-\left(\begin{array}{l} V\left(\omega \right)\\ V^{*}\left(-\omega \right) \end{array}\right)$$ 23

where the matrix A usually contains single particle excitation frequencies along the diagonal and couplings among them in the off-diagonal blocks, whereas B couples excitations and de-excitations. X(ω) and Y(−ω) , respectively contain the Fourier transformed resonant and antiresonant transition amplitudes describing the first-order change in the system density matrix upon perturbation, while V(ω) and $V^{*}\left(-\omega \right)$ store matrix elements of the perturbation.

By comparing eq 16 with eq 23 we can achieve quantization of the classical PCM-NP model with generic dielectric function. This is done by mapping the $A_{\theta }$ and $B_{\theta }$ matrices to the A and B blocks of the linear response matrix of a quantum system featuring single particle plasmonic excitation frequencies ${\mathrm{\omega ̅}}_{p}+\lambda_{\theta }\frac{A_{p}}{4\pi {\mathrm{\omega ̅}}_{p}}$ and damping rates γ_*p*_. In turn, $V_{\theta }\left(\omega \right)$ can be identified with the external perturbation and therefore $q_{\theta }^{R}\left(\omega \right)$ with X(ω) and $q_{\theta }^{A}\left(-\omega \right)$ with Y(−ω) . In other words, the classical macroscopic polarization eq (eq 1) of the NP can be exactly mapped to the linear response polarization of a quantum system composed of a set of coupled and damped quantum plasmon modes for each geometric BEM eigenmode θ of the NP, independently. This represents the central conceptual contribution of the present work, demonstrating that the classical PCM-NP formalism with a generic dielectric function can be naturally mapped onto the linear response of a quantum system through a direct comparison with the standard linear-response equations of TDDFT or TDHF.^42–45^

By following standard response theory^42,44^ (see SI 1 for details), for each θ we can solve the generalized eigenvalue problem associated with eq 23:

$$\left(\begin{array}{ll} A_{\theta } & B_{\theta }\\ B_{\theta }^{*} & A_{\theta }^{*} \end{array}\right)U_{\theta }=\left(\begin{array}{ll} I & 0\\ 0 & -I \end{array}\right)U_{\theta }d_{\theta }$$ 24

where ***d***_θ_ is the diagonal eigenvalue matrix with elements $d_{\theta ,n}=\omega_{\theta ,n}-i\frac{\gamma_{\theta ,n}}{2}$ and $d_{\theta ,-n}=-\omega_{\theta ,n}-i\frac{\gamma_{\theta ,n}}{2}$ for resonant and antiresonant transitions, respectively. ***U***_θ_ collects in its columns the corresponding generalized eigenvectors. Their properties are recalled in SI 1. From eq 24 we can identify ω_θ,*n*_ with the true excitation energy of the quantum NP plasmonic state |θ,*n*⟩ with decay rate $\gamma_{\theta ,n}$ for each BEM eigenmode θ. This leads to the following NP plasmonic Hamiltonian,

$${Ĥ}_{NP}=\underset{\theta ,n}{\sum }\left(\omega_{\theta ,n}-i\frac{\gamma_{\theta ,n}}{2}\right){\mathrm{b̂}}_{\theta ,n}^{†}{\mathrm{b̂}}_{\theta ,n}$$ 25

where *b̂*_θ,*n*_^†^ and *b̂*_θ,*n*_ are the corresponding bosonic creation and annihilation operators.^34,35,46^ Such a Hamiltonian can be coupled to any quantum chemistry molecular description as discussed below.

Moreover, we can also identify within this picture transition elements of relevant quantum operators. Focusing on surface charges, similarly to ref 35, upon introducing the quantized surface charge operator for the *k*-th surface tessera *q̂*_*k*_, the quantum transition charges for each coupled |θ,*p*⟩ state can be identified:

$$\left\langle 0\right|{\mathrm{q̂}}_{k}\left|\theta ,p\right\rangle ={\left(S^{-1/2}T\right)}_{k\theta }\sqrt{\frac{A_{p}}{2{\mathrm{\omega ̅}}_{p}}\left(1+\frac{\lambda_{\theta }}{2\pi }\right)}$$ 26

leading in turn to

$$\left\langle 0\right|{\mathrm{q̂}}_{k}\left|\theta ,n\right\rangle =\underset{p}{\sum }{\left(S^{-1/2}T\right)}_{k\theta }\left[\sqrt{\frac{A_{p}}{2{\mathrm{\omega ̅}}_{p}}\left(1+\frac{\lambda_{\theta }}{2\pi }\right)}X_{\theta ,pn}+{\left(\sqrt{\frac{A_{p}}{2{\mathrm{\omega ̅}}_{p}}\left(1+\frac{\lambda_{\theta }}{2\pi }\right)}\right)}^{*}Y_{\theta ,pn}\right]$$ 27

The quantities defined in eqs 26 and 27 are transition matrix elements of the surface charge operator *q̂*, which is a quantum operator in this formalism.^47,48^ *X*_θ,*pn*_ and Y_θ,*pn*_ are elements of the *n*-th column of the ***U***_θ_ matrix

Based on eqs 27 and response theory^42,44^ (SI 1 for details), the dipole–dipole polarizability tensor α_*ab*_(ω) of the quantum NP can be expressed in its spectral form as

$$\alpha_{ab}\left(\omega \right)=\underset{\theta ,n}{\sum }\frac{\left\langle 0\right|{\mathrm{\mu ̂}}_{a}\left|\theta ,n\right\rangle \left\langle \theta ,n\right|{\mathrm{\mu ̂}}_{b}\left|0\right\rangle }{\omega_{\theta ,n}-\omega -i\frac{\gamma_{\theta ,n}}{2}}+\frac{{\left\langle 0\right|{\mathrm{\mu ̂}}_{a}\left|\theta ,n\right\rangle }^{*}{\left\langle \theta ,n\right|{\mathrm{\mu ̂}}_{b}\left|0\right\rangle }^{*}}{\omega_{\theta ,n}+\omega +i\frac{\gamma_{\theta ,n}}{2}}$$ 28

where *μ̂*_*a*_ is defined as ${\mathrm{\mu ̂}}_{a}=\underset{k}{\sum }{\mathrm{q̂}}_{k}{\mathrm{r⃗}}_{k,a}$ with ${\mathrm{r⃗}}_{k,a}$ being the *a*th component of the position vector pointing to the *k*th NP tessera. Equation 28 holds for all positive *A*_*p*_. A slightly more complex expression is obtained in the general case (see the Supporting Information).

We stress that the plasmonic states so introduced should be regarded as effective states that describe the more complex electronic structure of the true metal NP, featuring quasi-continuum bands, and that they do not constitute exact eigenstates of any unperturbed Hamiltonian. If this were the case, the $B_{\theta }$ block would be exactly zero according to response theory from exact states.^44^ This is clearly not the case for a finite NP, where $B_{\theta }$ is nonvanishing. Interestingly, for an infinite planar plasmonic surface λ_θ_ would be zero,^49^ making the coupling blocks vanishing.

The theory presented above has been numerically validated on a test case NP of ellipsoidal shape (Figure 1) either made of silver or gold. These two widely used plasmonic metals are well-known to exhibit multiple interband transitions that cannot be captured by a simple DL dielectric function model, thereby making a generic dielectric function approach desirable in these cases. The corresponding function *f*(ω) (eq 9) has been fitted to data from ref 50 for Ag and ref 51 for Au. The fitted pole parameters are reported in the computational details section (SI 3). In Figure 2 the imaginary part of the NP polarizability, which is proportional to the NP absorption cross-section, is shown for both silver (Figure 2a) and gold (Figure 2b). The classical result (solid blue) is obtained from the induced dipole moment of the NP upon numerical solution of eq 1, while the quantum result (dashed orange) is computed according to eq 28. The driving field is oriented along the main axis of the NP (*x* axis) and the α_*xx*_(ω) component of the polarizability tensor is analyzed. Since in the quasi-static limit only dipolar modes contribute to the NP optical response, only the BEM dipolar eigenmode along the *x* axis is considered in Figure 2 (see also SI 3). Figure 2 clearly shows that the here introduced quantum states of the NP correctly reproduce the classical macroscopic polarization of the NP in both cases, thus validating the quantization procedure detailed above. The real part of the polarizability is also correctly reproduced, as shown in Figure S3. The implementation has also been tested on a gold spherical NP (Figure S4), confirming the robustness of the method against different NP shapes.

**Figure 1:**
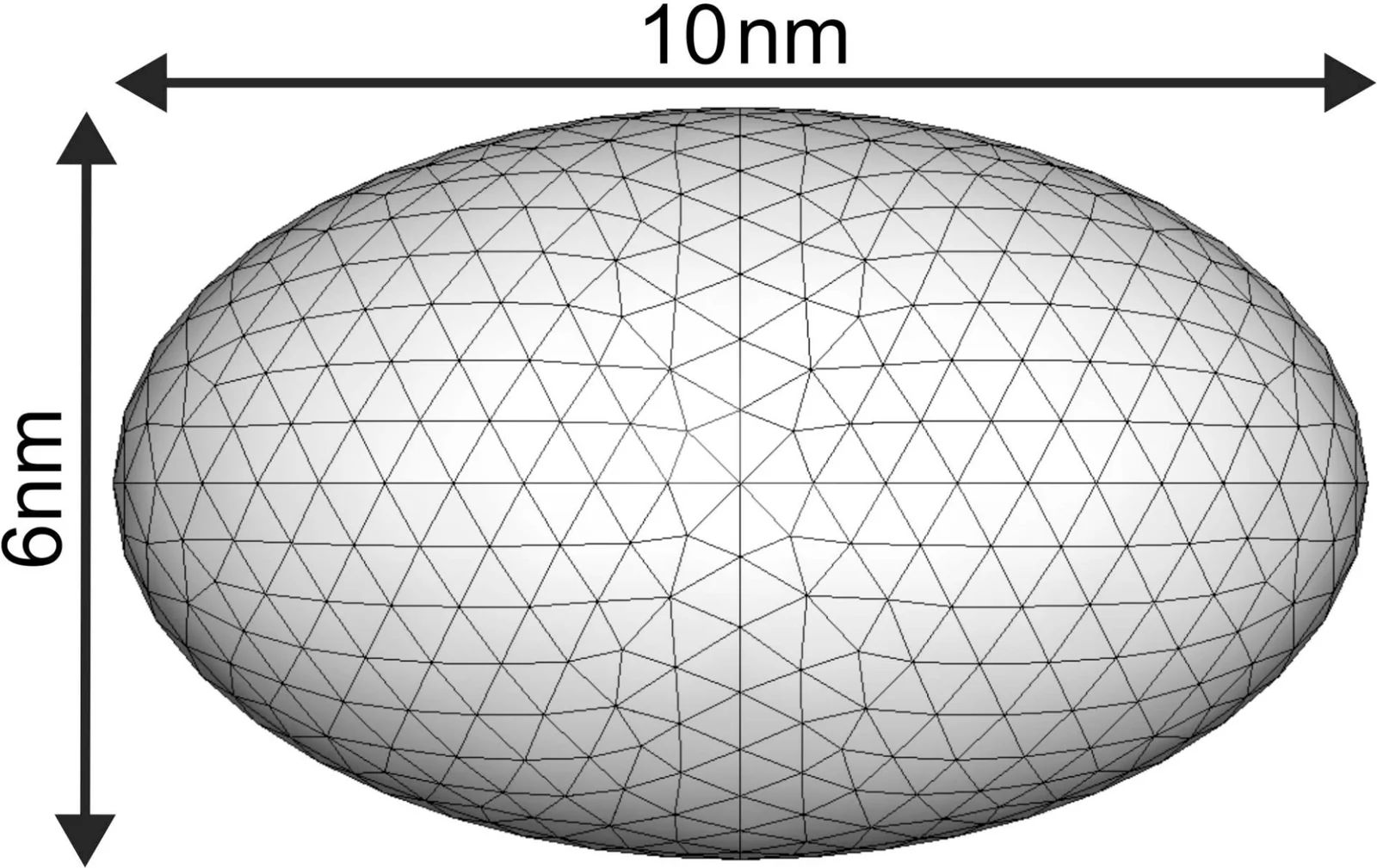
Ellipsoidal NP model used for simulations featuring 1371 surface tesserae.

**Figure 2:**
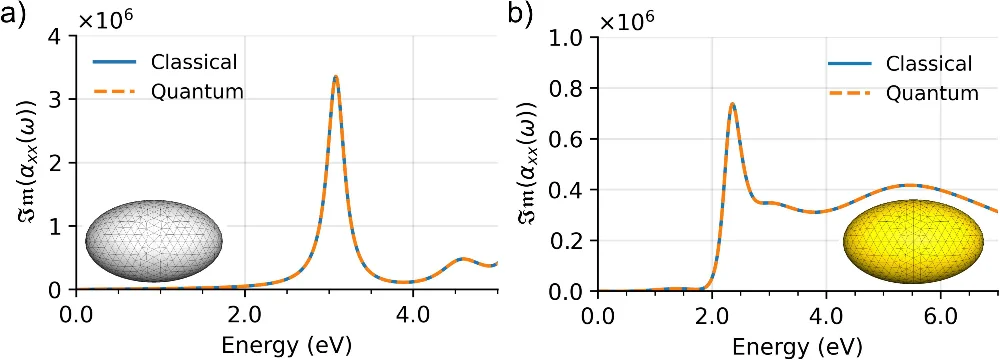
Imaginary part of the *xx* component of the dipole–dipole polarizability tensor for the ellipsoidal NP (Figure 1) when the *f*(ω) function is fitted to Ag Brendel-Bormann^50^ (a) or Au Etchegoin^51^ (b) reference data. The classical PCM-NP (solid blue) and quantum Q-PCM-NP (dashed orange) results are shown.

The quantum mode description introduced above can be straightforwardly coupled to a quantum-chemical molecular description by extending the procedure of ref 35, originally developed for the simpler DL case. Following ref 35 and eq 25, the full plasmon–molecule Hamiltonian describing the interaction between a molecule and the NP quantum modes derived from the general dielectric function can be written as (SI 2),

$${Ĥ}_{\mathrm{tot}}={Ĥ}_{\mathrm{mol}}+{Ĥ}_{\mathrm{NP}}+{Ĥ}_{\mathrm{int}}={Ĥ}_{\mathrm{mol}}+\underset{\theta ,n}{\sum }\left(\omega_{\theta ,n}-i\frac{\gamma_{\theta ,n}}{2}\right){\mathrm{b̂}}_{\theta ,n}^{†}{\mathrm{b̂}}_{\theta ,n}+\underset{\theta ,n,k}{\sum }{\mathrm{V̂}}_{k}\left(\langle \theta ,n|{\mathrm{q̂}}_{k}\left|0\right\rangle {\mathrm{b̂}}_{\theta ,n}^{†}+\langle 0|{\mathrm{q̂}}_{k}\left|\theta ,n\right\rangle {\mathrm{b̂}}_{\theta ,n}\right)$$ 29

where *Ĥ*_mol_ is the standard molecular electronic Hamitonian and *V̂̂*_*k*_ is the molecular electrostatic potential operator evaluated at the centroid of the *k*-th surface tessera of the NP.

The complete Hamiltonian in eq 29 can, in principle, be solved fully ab initio using QED–quantum chemistry methods to accurately capture electron–plasmon correlation effects, as demonstrated in ref 35. However, this approach is often computationally demanding due to the additional plasmonic degrees of freedom beyond the already costly electronic structure treatment. A viable strategy to reduce this cost is to first calculate a given number of electronic states of the isolated molecule and subsequently couple them to the plasmonic modes, as in ref 29. This alternative is, in principle, exact in the limit where all plasmonic modes of the NP are included, thereby recovering the full configuration-interaction limit. Consequently, truncating the plasmonic space to a finite subset of modes is an approximation, leading to the neglect of part of the plasmon–molecule correlation.

The primary focus of the present work is to introduce a novel quantization scheme for the NP response that is applicable to general dielectric functions rather than being restricted to a simple DL oscillator model. Accordingly, solving eq 29 at fully ab initio level using QED ab initio methods, as in ref 35, lies beyond the scope of the present study. Nevertheless, the quantum plasmonic modes derived here can be readily incorporated into such an approach.

In the following, we adopt the above described framework for a proof-of-concept application. Specifically, the molecular Hamiltonian is first solved independently, after which it is coupled to a truncated set of quantum plasmonic modes. Therefore, starting from eq 29 and considering the molecular eigenstates, |*g*⟩ (for the ground state) and |*e*⟩ (for a general excited state), *Ĥ*_tot_ in the rotating wave approximation can be expressed as (SI 2 for details)

$${Ĥ}_{\mathrm{tot}}=\omega_{g}\left|g\right\rangle \left\langle g\right|+\underset{e}{\sum }\left(\omega_{e}-i\frac{\gamma_{e}}{2}\right)\left|e\right\rangle \left\langle e\right|+\underset{\theta ,n}{\sum }\left(\omega_{\theta ,n}-i\frac{\gamma_{\theta ,n}}{2}\right){\mathrm{b̂}}_{\theta ,n}^{†}{\mathrm{b̂}}_{\theta ,n}++\underset{\theta ,n,k}{\sum }\left({\mathrm{V̂}}_{k}^{gg}\left|g\right\rangle \langle g|+\underset{e}{\sum }{\mathrm{V̂}}_{k}^{ee}\left|e\right\rangle \langle e|\right)\left(\langle \theta ,n|{\mathrm{q̂}}_{k}\left|0\right\rangle {\mathrm{b̂}}_{\theta ,n}^{†}\;+\langle 0|{\mathrm{q̂}}_{k}\left|\theta ,n\right\rangle {\mathrm{b̂}}_{\theta ,n}\right)+\underset{\theta ,n,k,e}{\sum }{\mathrm{V̂}}_{k}^{eg}\left(\langle \theta ,n|{\mathrm{q̂}}_{k}\left|0\right\rangle {\mathrm{b̂}}_{\theta ,n}^{†}\left|g\right\rangle \langle e|\;+\langle 0|{\mathrm{q̂}}_{k}\left|\theta ,n\right\rangle {\mathrm{b̂}}_{\theta ,n}\left|e\right\rangle \langle g|\right)+\underset{\theta ,n,k,e<e^{\prime }}{\sum }{\mathrm{V̂}}_{k}^{ee^{\prime }}\left(\langle \theta ,n|{\mathrm{q̂}}_{k}\left|0\right\rangle {\mathrm{b̂}}_{\theta ,n}^{†}\left|e\right\rangle \langle e^{\prime }|+\langle 0|{\mathrm{q̂}}_{k}\left|\theta ,n\right\rangle {\mathrm{b̂}}_{\theta ,n}\left|e^{\prime }\right\rangle \langle e|\right)$$ 30

In eq 30 ω_*g*_ and ω_*e*_ are the molecular energies of ground and excited states, respectively, while γ_*e*_ is a phenomenological damping rate to account for the finite lifetime of the molecular excited states. Furthermore, *V̂^_k_gg^, V̂*_*k*_^*ee*^ and *V̂*_*k*_^*eg*^ are shorthand notations for the matrix elements ⟨*g*|*V̂*_*k*_|*g*⟩, ⟨*e*|*V̂*_*k*_|*e*⟩ and ⟨*e*|*V̂*_*k*_|*g*⟩. The first term accounts for NP–molecule ground-state polarization effects, the second for NP–molecule excited-state polarization effects whereas the third term *V̂*_*k*_^*eg*^, which is the electrostatic potential on the NP surface due to the molecular transition |*g*⟩ → |*e*⟩, is responsible for Jaynes–Cummings-like coupling terms. Additionally, the terms present in the last line of eq 30 promote a mixing of the bare molecular excited states due to molecule–NP polarization effects. Note that the term containing V^_k_gg^ arises because a realistic molecule generally possesses a nonzero permanent dipole moment already in its ground state. This permanent dipole polarizes the nearby NP. This contribution should not be confused with the ground-state dressing arising from the counter-rotating-wave terms in the light–matter coupling, discussed below.

In the present example the Hamiltonian of eq 30 is diagonalized in the single-excitation subspace, |*g*⟩⊗|0_θ,*n*_⟩, |*e*⟩⊗|0_θ,*n*_⟩, |*g*⟩⊗|1_θ,*n*_⟩ where the notation |*g*⟩⊗|1_θ,*n*_⟩ means that the molecule is in its ground state but the plasmon mode |θ, *n*⟩ is excited. The effect of including the second-excitation manifold, as well as the counter-rotating-wave terms, is negligible for the present application, as discussed in SI 2. Nevertheless, the methodology presented here can readily account for these contributions whenever they become relevant.

Diagonalization of the resulting non-Hermitian Hamiltonian leads to the dipole–dipole polarizability of the coupled molecule–NP system, analogously to eq 28. The real and imaginary parts of the eigenvalues of *Ĥ*_tot_ correspond to the absorption energies and damping rates of the plexcitonic states, respectively. The associated transition dipoles can be constructed from the left and right eigenvectors of *Ĥ*_tot_, using the molecular transition dipoles and those of the Q-PCM-NP modes (SI 1 and 2).

Such methodology has been tested to study the plasmon–molecule coupling effects between a silver ellipsoidal NP and a quinolone molecule described at CIS/6-31g* level of theory, using a setup similar to that of ref 29. The molecule is placed 0.8 nm far from the NP surface, as shown in Figure 3a. The ellipsoidal NP has the same size and Ag dielectric function as the model in Figures 1 and 2a but employs a refined surface tessellation in the region closer to the molecule to improve the numerical evaluation of plasmon–molecule coupling terms.

**Figure 3:**
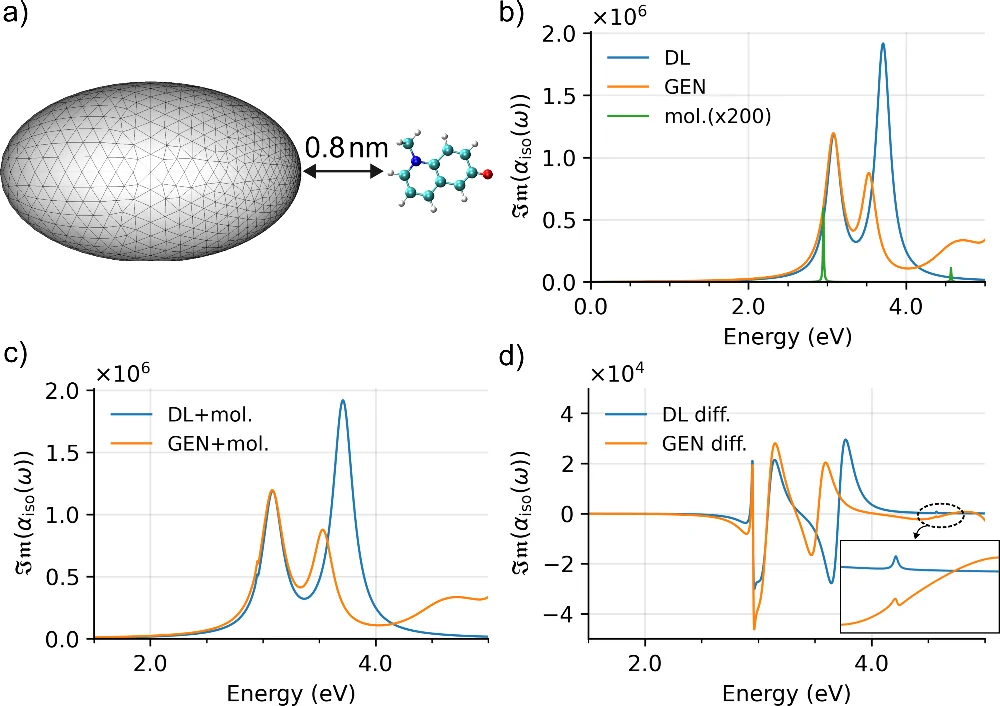
a) Setup used for investigating the quantum coupling between an ellipsoidal silver NP and one molecule (*N*-methyl-6-quinolone) described at the ab initio level. The molecule–NP dimensions are not to scale. The NP dimensions are the same of Figure 1 but more surface tesserae are considered to refine the region closer to the molecule. Overall 2122 surface tesserae are used. b) Imaginary part of the isotropic dipole–dipole polarizability for the ellipsoidal NP of panel a) when the *f*(ω) function is fitted to Ag Brendel–Bormann^50^ reference data (orange) or when a simpler DL model is used (blue). The DL parameters are chosen to reproduce the first dipolar plasmonic peak around 3.0 eV. The green line is the isotropic polarizability of the bare molecule scaled by a factor of 200. c) Corresponding polarizability plots of the coupled system (NP + molecule) when the generic dielectric function (orange) or DL quantum modes (blue) are used to investigate the molecule–NP coupling. d) Differences between the isotropic polarizability of the coupled system (panel c) and the corresponding bare plasmonic contribution shown in panel b for the two cases.

We note that this procedure does not account for the hybridization of the molecular and NP orbitals, which becomes relevant for few-angstrom NP–molecule distances. Such effects could be accounted for by explicitly treating a portion of the NP at fully quantum ab initio level together with the molecule, as done in, e.g., ref 52.

The imaginary part of the isotropic polarizability $\alpha_{\mathrm{iso}}=\frac{1}{3}\left(\alpha_{xx}+\alpha_{yy}+\alpha_{zz}\right)$ of the coupled system is investigated (Figure 3b–d) using either the general dielectric function (orange) or a single DL oscillator (blue), the latter relying on the simpler Q-PCM-NP scheme detailed in ref 35. In both cases, all plasmonic modes arising from the first 45 geometrical BEM eigenmodes have been considered to solve *Ĥ*_tot_ (SI 3 for more details). The DL parameters (SI 3) are tuned to reproduce the first plasmonic peak at ≈3.0 eV, which is oriented along the main axis of the NP. As shown by the isolated components (Figure 3a), the first two excited states of the quinolone molecule absorb at ≈2.95 eV and ≈ 4.57 eV and they are nearly resonant with the first and third plasmonic transitions predicted by the generic dielectric function (Figure 3b). Note also that the second plasmonic peak around ≈3.5, eV, present in both models although with different intensities, originates from the two transverse dipolar resonances, which contribute to the isotropic polarizability. Furthermore, the molecular transitions are considerably weaker than the plasmonic ones, approximately 40° tilted with respect to the NP main axis and the plasmon–molecule coupling between the brighest plasmon and the lowest excited-state of quinolone is ≈8 meV, thereby not particularly strong.

In the spectral region around 3.0 eV, where the two dielectric models show minimal differences, only minor variations are observed in the full-system spectrum (Figure 3c). These differences become more evident upon subtracting the corresponding bare plasmonic contribution from the polarizability of the coupled system, yielding the differential polarizability shown in Figure 3d, where the molecular contribution is evidenced. This representation reveals that near the plasmonic peak at 3.0 eV, both the DL and generic dielectric function models exhibit a similar sigmoidal-like behavior, consistent with plasmon–molecule coupling and so mixing. In contrast, more evident discrepancies emerge at higher energies. Around 3.5 eV, the DL model fails to capture the position and intensity of the transverse dipolar modes, leading to substantial deviations from the generic dielectric function prediction in the molecular contribution. This limitation stems from the fact that DL parameter tuning can reproduce only a single plasmonic mode at a time.

Additionally, in the region around 4.5 eV, the DL model displays a simple Lorentzian peak (blue line, see close-up), indicative of negligible mixing, whereas the generic dielectric function (orange line) manifests a more complex broadened structure arising from hybridization with plasmonic modes in this spectral region, including higher-order dark modes. The DL model fails to capture this effect due to its limited plasmonic mode content in this energy range and highlights a key limitation of the DL approach: its inherent restriction to describing properly a single plasmonic resonance at a time. As a result, they become inadequate when multiple plasmonic modes contribute to the overall optical response of the coupled system.

Additionally, it is worth emphasizing that the parameter tuning required to reproduce a given plasmonic peak (Figure 3a) with the DL dielectric function is system-specific and can be cumbersome, limiting the transferability of the approach. In contrast, the Q-PCM-NP framework based on a generic dielectric function provides a more robust and broadly applicable description of the plasmonic response, enabling a consistent treatment across different systems and spectral regions with no need for system-specific tuning procedures.

The Q-PCM-NP scheme with generic dielectric function introduced in this work can be readily applied to more complex plasmonic structures, such as scanning tunneling microscope tips and NP aggregates, previously treated within the simpler DL framework.^35,53^ The underlying theoretical framework remains unchanged. The current methodology relies on a quasistatic treatment of the NP electrodynamical problem. Extending it beyond that while quantizing the NP response and its coupling to a molecule may be possible, albeit theoretically involved, by combining macroscopic QED quantization schemes with the “dummy” surface approach introduced in refs 54 and 55 to account for retardation effects and radiative modes.

In this work, we have laid down an effective quantum mode description to model the optical response of arbitrarily shaped NPs endowed with empirical dielectric functions. The linear response polarization of the quantum NP correctly recovers the classical result obtained by solving the macroscopic Maxwell equations in the quasistatic limit via BEM. It is worth remarking that, although the NP linear response is typically well described by classical electrodynamics, its quantization becomes particularly relevant when the coupling with an external quantum emitter (i.e., a molecule) is investigated and specific phenomena, such as coupled electron–nuclear dynamics in the strong-coupling regime,^23,47^ few-emitter lasing,^20,21^ and strong-field driving,^28,29^ are targeted, to mention a few.

The developed approach can be straightforwardly integrated with QED ab initio theories.^56–58^ However, because of the presence of multiple plasmonic modes, effective-mode approaches^53^ are expected to be necessary for keeping the simulations computationally tractable.

Since BEM has extensively proven to be a convenient framework to model ab initio molecules in complex plasmonic environments,^22,34,53^ the development here presented paves the way for a fully quantum description of plexcitonic systems, involving NPs of arbitrary shape and made of realistic metals.

## Supplementary Material



## Notes

Detailed derivation of the Q-PCM-NP quantum modes with a generic dielectric function; coupling to a quantum chemistry molecular description; computational details; real part of polarizability for the ellipsoidal NP of Figure 1; results for a gold spherical NP; impact of the rotating wave approximation and the second excitation manifold (PDF)
