# Supplementary material for: Quantized Plasmon Modes for Metallic Nanoparticles of Arbitrary Shape with a Generic Dielectric Function
Source: J Phys Chem Lett. 2026 Aug 28;17(36):10429–36. doi: 10.1021/acs.jpclett.6c02006 (PMC13573622; doi:10.1021/acs.jpclett.6c02006)
Supplement: Supplementary file 1 [file jz6c02006_si_001.pdf]

Supplementary material for: Quantized plasmon modes for metallic nanoparticles of arbitrary shape with a generic dielectric function

Marco Romanelli,[†] Gabriel Gil,[‡,†] and Stefano Corni[*,†,¶,§]

†*Department of Chemical Sciences, University of Padova, via Marzolo 1, 35131 Padova, Italy*

‡*Dipartimento di Scienze e Innovazione Tecnologica, Università degli Studi del Piemonte Orientale Amedeo Avogadro, V.le Teresa Michel 11, 15121 Alessandria, Italy*

¶*CNR Institute of Nanoscience, via Campi 213/A, 41125 Modena, Italy*

§*Padua Quantum Technologies Research Center, University of Padova, 35131 Padova, Italy*

E-mail: stefano.corni@unipd.it

Contents

1 Q-PCM-NP with generic dielectric function **3**

2 Plasmon-molecule Hamiltonian in the generic dielectric function framework **12**

3 Computational details **14**

4 Real part of $\alpha_{xx}(\omega)$ for the ellipsoidal NP of Figs.1-2 **18**

5 Results for a gold spherical NP **19**

6 Impact of the rotating wave approximation and the second excitation manifold **20**

References **21**

1 Q-PCM-NP with generic dielectric function

The goal of this section is to manipulate the classical PCM-NP equation so to arrive to a format that is identical to the response equation of a quantum system.

Within the generic dielectric function approach,[1] the classical PCM-NP equations read

$$\boldsymbol{q}(\omega) = \frac{1}{2\pi} f(\omega) \boldsymbol{F}(\omega) \tag{S1}$$

with

$$\boldsymbol{F}(\omega) = -\left[\boldsymbol{A}\boldsymbol{D}^\dagger \boldsymbol{q}(\omega) + \boldsymbol{S}^{-1}\left(2\pi\mathbb{I} + \boldsymbol{D}\boldsymbol{A}\right)\boldsymbol{V}(\omega)\right] \tag{S2}$$

and

$$f(\omega) = \frac{\epsilon(\omega) - 1}{\epsilon(\omega) + 1} \approx \sum_p^N \frac{A_p}{\omega_p^2 - \omega^2 - i\gamma_p\omega}\,. \tag{S3}$$

As discussed in Ref.,[1] using Eq.S3 the classical response charges of Eq.S1 can be decomposed as pole-dependent charges as $\boldsymbol{q}(\omega) = \sum_p^N \boldsymbol{q}_p(\omega)$, turning Eq.S1 into

$$\frac{2\pi}{A_p}(\omega_p^2 - \omega^2 - i\gamma_p\omega)\boldsymbol{q}_p(\omega) = -\left[\boldsymbol{A}\boldsymbol{D}^\dagger \boldsymbol{q}(\omega) + \boldsymbol{S}^{-1}\left(2\pi\mathbb{I} + \boldsymbol{D}\boldsymbol{A}\right)\boldsymbol{V}(\omega)\right]. \tag{S4}$$

To achieve quantization (Q-PCM-NP) it is more convenient to explicitly express the resonant and anti-resonant contributions hidden in Eq.S3 as

$$f(\omega) \approx \sum_p^N \frac{A_p}{\omega_p^2 - \omega^2 - i\gamma_p\omega} = \sum_p^N \frac{A_p}{2\overline{\omega}_p}\left(\frac{1}{\overline{\omega}_p - \omega - i\gamma_p/2} + \frac{1}{\overline{\omega}_p + \omega + i\gamma_p/2}\right) \tag{S5}$$

with

$$\overline{\omega}_p = \sqrt{\omega_p^2 - \gamma_p^2/4}. \tag{S6}$$

The first term in round brackets of Eq.S5 can be defined as the resonant term (R, $\propto (\overline{\omega}_p - \omega)^{-1}$) while the other one is the anti-resonant (A, $\propto (\overline{\omega}_p + \omega)^{-1}$). Consequently, the classical response charges can be further decomposed as $\boldsymbol{q}(\omega) = \sum_p^N \boldsymbol{q}_p(\omega) = \sum_p^N \left(\boldsymbol{q}_p^R(\omega) + \boldsymbol{q}_p^A(\omega)\right)$,

which turns Eq.S4 into two separate coupled equations for each pth pole,

$$\begin{aligned}
\frac{4\pi\overline{\omega}_p}{A_p}(\overline{\omega}_p-\omega-i\frac{\gamma_p}{2})\boldsymbol{q}_p^R(\omega) &= -\big[\boldsymbol{A}\boldsymbol{D}^\dagger\sum_{p'}^{N}\left(\boldsymbol{q}_{p'}^R(\omega)+\boldsymbol{q}_{p'}^A(\omega)\right)+\boldsymbol{S}^{-1}\left(2\pi\mathbb{I}+\boldsymbol{D}\boldsymbol{A}\right)\boldsymbol{V}(\omega)\big]\,,\\
\frac{4\pi\overline{\omega}_p}{A_p}(\overline{\omega}_p+\omega+i\frac{\gamma_p}{2})\boldsymbol{q}_p^A(\omega) &= -\big[\boldsymbol{A}\boldsymbol{D}^\dagger\sum_{p'}^{N}\left(\boldsymbol{q}_{p'}^R(\omega)+\boldsymbol{q}_{p'}^A(\omega)\right)+\boldsymbol{S}^{-1}\left(2\pi\mathbb{I}+\boldsymbol{D}\boldsymbol{A}\right)\boldsymbol{V}(\omega)\big]\,.
\end{aligned} \tag{S7}$$

Furthermore, by turning the BEM kernel in diagonal form as shown in Refs.[2,3] via the eigenmode decomposition ($\boldsymbol{S}^{-1/2}\boldsymbol{D}\boldsymbol{A}\boldsymbol{S}^{1/2}=\boldsymbol{T}\boldsymbol{\lambda}\boldsymbol{T}^\dagger$) and by left-multiplying each Eq.S7 by $\boldsymbol{S}$ (while considering the following relations[3] $\boldsymbol{S}=\boldsymbol{S}^{\frac{1}{2}}\boldsymbol{S}^{\frac{1}{2}}$, $\boldsymbol{S}\boldsymbol{A}\boldsymbol{D}^\dagger=\boldsymbol{D}\boldsymbol{A}\boldsymbol{S}$), straightforward matrix algebra leads to

$$\begin{aligned}
&\left(\boldsymbol{S}^{1/2}\boldsymbol{T}\boldsymbol{K}_{pp}^R(\omega)\boldsymbol{T}^\dagger\boldsymbol{S}^{1/2}\right)\boldsymbol{q}_p^R(\omega)+\sum_{p'\neq p}^{N}\left(\boldsymbol{S}^{1/2}\boldsymbol{T}\tilde{\boldsymbol{\lambda}}_{pp'}\boldsymbol{T}^\dagger\boldsymbol{S}^{1/2}\right)\boldsymbol{q}_{p'}^R(\omega)+\\
&\qquad\sum_{p'}^{N}\left(\boldsymbol{S}^{1/2}\boldsymbol{T}\tilde{\boldsymbol{\lambda}}_{pp'}\boldsymbol{T}^\dagger\boldsymbol{S}^{1/2}\right)\boldsymbol{q}_{p'}^A(\omega)=-\boldsymbol{V}(\omega)\,,\\
&\left(\boldsymbol{S}^{1/2}\boldsymbol{T}\boldsymbol{K}_{pp}^A(\omega)\boldsymbol{T}^\dagger\boldsymbol{S}^{1/2}\right)\boldsymbol{q}_p^A(\omega)+\sum_{p'\neq p}^{N}\left(\boldsymbol{S}^{1/2}\boldsymbol{T}\tilde{\boldsymbol{\lambda}}_{pp'}\boldsymbol{T}^\dagger\boldsymbol{S}^{1/2}\right)\boldsymbol{q}_{p'}^A(\omega)+\\
&\qquad\sum_{p'}^{N}\left(\boldsymbol{S}^{1/2}\boldsymbol{T}\tilde{\boldsymbol{\lambda}}_{pp'}\boldsymbol{T}^\dagger\boldsymbol{S}^{1/2}\right)\boldsymbol{q}_{p'}^R(\omega)=-\boldsymbol{V}(\omega)
\end{aligned} \tag{S8}$$

with

$$\begin{aligned}
K_{pp,\theta\theta}^R(\omega) &= \frac{\frac{4\pi\overline{\omega}_p}{A_p}(\overline{\omega}_p-\omega-i\frac{\gamma_p}{2})+\lambda_\theta}{2\pi+\lambda_\theta}=\frac{\overline{\omega}_p-\omega-i\frac{\gamma_p}{2}+\lambda_\theta\frac{A_p}{4\pi\overline{\omega}_p}}{\frac{A_p}{2\overline{\omega}_p}\left(1+\frac{\lambda_\theta}{2\pi}\right)}\,,\\
K_{pp,\theta\theta}^A(\omega) &= \frac{\frac{4\pi\overline{\omega}_p}{A_p}(\overline{\omega}_p+\omega+i\frac{\gamma_p}{2})+\lambda_\theta}{2\pi+\lambda_\theta}=\frac{\overline{\omega}_p+\omega+i\frac{\gamma_p}{2}+\lambda_\theta\frac{A_p}{4\pi\overline{\omega}_p}}{\frac{A_p}{2\overline{\omega}_p}\left(1+\frac{\lambda_\theta}{2\pi}\right)}\,,\\
\tilde{\lambda}_{pp',\theta\theta} &= \frac{\lambda_\theta}{2\pi+\lambda_\theta}\,.
\end{aligned} \tag{S9}$$

Note that the matrices of Eq.S9 are diagonal in $\theta\theta'$ and the absence of pp' indexes on the right-hand side of $\tilde{\lambda}_{pp',\theta\theta}=\frac{\lambda_\theta}{2\pi+\lambda_\theta}$ means that these coupling matrix elements do not depend

on the pole indexes, but only on the BEM eigenmode one (θ). Note that since the matrix $\boldsymbol{S}^{-1/2}\boldsymbol{DAS}^{1/2}$ is real and symmetric, the λ_θ eigenvalues are also all real.

Making explicit matrix multiplication, Eq.S8 reads

$$
\begin{aligned}
&\sum_{\theta k}\left(S^{1/2}T\right)_{j\theta} K^R_{pp,\theta\theta}\left(T^\dagger S^{1/2}\right)_{\theta k} q^R_{pk} + \sum_{\theta k}\sum_{p'\neq p}^{N}\left(S^{1/2}T\right)_{j\theta}\tilde{\lambda}_{pp',\theta\theta}\left(T^\dagger S^{1/2}\right)_{\theta k} q^R_{p'k} + \\
&\sum_{\theta k}\sum_{p'}^{N}\left(S^{1/2}T\right)_{j\theta}\tilde{\lambda}_{pp',\theta\theta}\left(T^\dagger S^{1/2}\right)_{\theta k} q^A_{p'k} = -V_j\,, \\
&\sum_{\theta k}\left(S^{1/2}T\right)_{j\theta} K^A_{pp,\theta\theta}\left(T^\dagger S^{1/2}\right)_{\theta k} q^A_{pk} + \sum_{\theta k}\sum_{p'\neq p}^{N}\left(S^{1/2}T\right)_{j\theta}\tilde{\lambda}_{pp',\theta\theta}\left(T^\dagger S^{1/2}\right)_{\theta k} q^A_{p'k} + \\
&\sum_{\theta k}\sum_{p'}^{N}\left(S^{1/2}T\right)_{j\theta}\tilde{\lambda}_{pp',\theta\theta}\left(T^\dagger S^{1/2}\right)_{\theta k} q^R_{p'k} = -V_j
\end{aligned}
\tag{S10}
$$

where the (ω) have been dropped to ease notation and $q^{R/A}_{pk} = q^{R/A}_{pk}(\omega)$ represents the kth surface classical resonant/anti-resonant response charge due to the pth pole. $V_j = V_j(\omega)$ is instead the external potential acting on the jth surface tessera.

Upon defining $\tilde{q}^R_{\theta p} = \sum_k \left(T^\dagger S^{1/2}\right)_{\theta k} q^R_{pk}$, $\tilde{q}^A_{\theta p} = \sum_k \left(T^\dagger S^{1/2}\right)_{\theta k} q^A_{pk}$ and $\tilde{V}_{\theta p} = \sum_j \left(T^\dagger S^{-1/2}\right)_{\theta j} V_j$ Eq.S10 can be recast as

$$
\begin{aligned}
K^R_{pp,\theta\theta}\tilde{q}^R_{\theta p} + \sum_{p'\neq p}^{N}\tilde{\lambda}_{pp',\theta\theta}\tilde{q}^R_{\theta p'} + \sum_{p'}^{N}\tilde{\lambda}_{pp',\theta\theta}\tilde{q}^A_{\theta p'} = -\tilde{V}_{\theta p}\,, \\
K^A_{pp,\theta\theta}\tilde{q}^A_{\theta p} + \sum_{p'\neq p}^{N}\tilde{\lambda}_{pp',\theta\theta}\tilde{q}^A_{\theta p'} + \sum_{p'}^{N}\tilde{\lambda}_{pp',\theta\theta}\tilde{q}^R_{\theta p'} = -\tilde{V}_{\theta p}\,.
\end{aligned}
\tag{S11}
$$

Note that we have added a p subscript to the potential $\tilde{V}$ to make the terms of the equations uniform, but that $\tilde{V}_{\theta p}$ is actually independent from p.

Further manipulation of Eq.S11 finally leads to

$$\begin{aligned}
&\left(\overline{\omega}_p - i\frac{\gamma_p}{2} + \lambda_\theta \frac{A_p}{4\pi\overline{\omega}_p}\right)\mathbb{q}^R_{\theta p} - \omega \mathbb{q}^R_{\theta p} + \sum_{p'\neq p}^{N} \sqrt{\frac{A_p}{2\overline{\omega}_p}\left(1+\frac{\lambda_\theta}{2\pi}\right)}\tilde{\lambda}_{pp',\theta\theta}\sqrt{\frac{A_{p'}}{2\overline{\omega}_{p'}}\left(1+\frac{\lambda_\theta}{2\pi}\right)}\mathbb{q}^R_{\theta p'} + \\
&\sum_{p'}^{N} \sqrt{\frac{A_p}{2\overline{\omega}_p}\left(1+\frac{\lambda_\theta}{2\pi}\right)}\tilde{\lambda}_{pp',\theta\theta}\left(\sqrt{\frac{A_{p'}}{2\overline{\omega}_{p'}}\left(1+\frac{\lambda_\theta}{2\pi}\right)}\right)^* \mathbb{q}^A_{\theta p'} = -\mathbb{V}_{\theta p}\,, \\
&\left(\overline{\omega}_p + i\frac{\gamma_p}{2} + \lambda_\theta \frac{A_p}{4\pi\overline{\omega}_p}\right)\mathbb{q}^A_{\theta p} + \omega \mathbb{q}^A_{\theta p} + \sum_{p'\neq p}^{N} \left(\sqrt{\frac{A_p}{2\overline{\omega}_p}\left(1+\frac{\lambda_\theta}{2\pi}\right)}\right)^*\tilde{\lambda}_{pp',\theta\theta}\left(\sqrt{\frac{A_{p'}}{2\overline{\omega}_{p'}}\left(1+\frac{\lambda_\theta}{2\pi}\right)}\right)^* \\
&\mathbb{q}^A_{\theta p'} + \sum_{p'}^{N} \left(\sqrt{\frac{A_p}{2\overline{\omega}_p}\left(1+\frac{\lambda_\theta}{2\pi}\right)}\right)^*\tilde{\lambda}_{pp',\theta\theta}\left(\sqrt{\frac{A_{p'}}{2\overline{\omega}_{p'}}\left(1+\frac{\lambda_\theta}{2\pi}\right)}\right)\mathbb{q}^R_{\theta p'} = -\mathbb{V}^*_{\theta p}\,,
\end{aligned} \tag{S12}$$

where

$$\begin{aligned}
\mathbb{q}^R_{\theta p} &= \frac{1}{\sqrt{\frac{A_p}{2\overline{\omega}_p}\left(1+\frac{\lambda_\theta}{2\pi}\right)}}\tilde{q}^R_{\theta p} \\
\mathbb{q}^A_{\theta p} &= \frac{1}{\left(\sqrt{\frac{A_p}{2\overline{\omega}_p}\left(1+\frac{\lambda_\theta}{2\pi}\right)}\right)^*}\tilde{q}^A_{\theta p} \\
\mathbb{V}_{\theta p} &= \mathrm{sgn}(A_p)\left(\sqrt{\frac{A_p}{2\overline{\omega}_p}\left(1+\frac{\lambda_\theta}{2\pi}\right)}\right)^*\tilde{V}_{\theta p} \\
\mathbb{V}^*_{\theta p} &= \mathrm{sgn}(A_p)\sqrt{\frac{A_p}{2\overline{\omega}_p}\left(1+\frac{\lambda_\theta}{2\pi}\right)}\tilde{V}_{\theta p}\,.
\end{aligned} \tag{S13}$$

The $\mathrm{sgn}(A_p)$ in the last two equations is needed to take into account that some A_p may be negative, as described and explained in ref.[1] In practice, we are translating this into a modified perturbation whose matrix elements have a change of sign for those $|\theta, p\rangle$ that corresponds to negative A_p.

Recalling the form of $\tilde{\lambda}_{pp',\theta\theta}$ (Eq.S9) Eq.S12 can be further simplified to

$$\begin{aligned}
&\left(\overline{\omega}_p - i\frac{\gamma_p}{2} + \lambda_\theta \frac{A_p}{4\pi\overline{\omega}_p}\right)\mathbb{q}^R_{\theta p} - \omega \mathbb{q}^R_{\theta p} + \sum_{p'\neq p}^{N}\sqrt{\frac{A_p}{2\overline{\omega}_p}}\frac{\lambda_\theta}{2\pi}\sqrt{\frac{A_{p'}}{2\overline{\omega}_{p'}}}\mathbb{q}^R_{\theta p'} + \\
&\sum_{p'}^{N}\sqrt{\frac{A_p}{2\overline{\omega}_p}}\frac{\lambda_\theta}{2\pi}\left(\sqrt{\frac{A_{p'}}{2\overline{\omega}_{p'}}}\right)^*\mathbb{q}^A_{\theta p'} = -\mathbb{V}_{\theta p}\,, \\
&\left(\overline{\omega}_p + i\frac{\gamma_p}{2} + \lambda_\theta \frac{A_p}{4\pi\overline{\omega}_p}\right)\mathbb{q}^A_{\theta p} + \omega \mathbb{q}^A_{\theta p} + \sum_{p'\neq p}^{N}\left(\sqrt{\frac{A_p}{2\overline{\omega}_p}}\right)^*\frac{\lambda_\theta}{2\pi}\left(\sqrt{\frac{A_{p'}}{2\overline{\omega}_{p'}}}\right)^*\mathbb{q}^A_{\theta p'} + \\
&\sum_{p'}^{N}\left(\sqrt{\frac{A_p}{2\overline{\omega}_p}}\right)^*\frac{\lambda_\theta}{2\pi}\left(\sqrt{\frac{A_{p'}}{2\overline{\omega}_{p'}}}\right)\mathbb{q}^R_{\theta p'} = -\mathbb{V}^*_{\theta p}\,.
\end{aligned} \tag{S14}$$

Basically, for each θth BEM eigenmode Eq.S14 corresponds to an independent matrix equation of the following form

$$\left[\begin{pmatrix} \mathbb{A}_\theta & \mathbb{B}_\theta \\ \mathbb{B}^*_\theta & \mathbb{A}^*_\theta \end{pmatrix} - \omega \begin{pmatrix} \mathbb{I} & 0 \\ 0 & -\mathbb{I} \end{pmatrix}\right]\begin{pmatrix} \mathbb{q}^R_\theta \\ \mathbb{q}^A_\theta \end{pmatrix} = -\begin{pmatrix} \mathbb{V}_\theta \\ \mathbb{V}^*_\theta \end{pmatrix} \tag{S15}$$

with

$$\begin{aligned}
(\mathbb{A}_\theta)_{pp'} &= \left(\overline{\omega}_p - i\frac{\gamma_p}{2} + \lambda_\theta \frac{A_p}{4\pi\overline{\omega}_p}\right)\delta_{pp'} + (1-\delta_{pp'})\sqrt{\frac{A_p}{2\overline{\omega}_p}}\frac{\lambda_\theta}{2\pi}\sqrt{\frac{A_{p'}}{2\overline{\omega}_{p'}}} \\
(\mathbb{B}_\theta)_{pp'} &= \sqrt{\frac{A_p}{2\overline{\omega}_p}}\frac{\lambda_\theta}{2\pi}\left(\sqrt{\frac{A_{p'}}{2\overline{\omega}_{p'}}}\right)^*
\end{aligned} \tag{S16}$$

and $\mathbb{q}^{R/A}_\theta$,$\mathbb{V}_\theta$ are vectors of dimension N (n. of poles in Eq.S3) which store the corresponding pth elements $\mathbb{q}^{R/A}_{\theta,p}$, $\mathbb{V}_{\theta,p}$.

To give a graphical illustration of the matrices $\mathbb{A}_\theta$, $\mathbb{B}_\theta$, in the hypothetical case of 3 poles (N=3), the matrices would look

$$
\begin{aligned}
\mathbb{A}_\theta &= \begin{pmatrix} \overline{\overline{K}}_{11,\theta\theta} & \overline{\overline{\lambda}}_{12,\theta\theta} & \overline{\overline{\lambda}}_{13,\theta\theta} \\ \overline{\overline{\lambda}}_{12,\theta\theta} & \overline{\overline{K}}_{22,\theta\theta} & \overline{\overline{\lambda}}_{23,\theta\theta} \\ \overline{\overline{\lambda}}_{13,\theta\theta} & \overline{\overline{\lambda}}_{23,\theta\theta} & \overline{\overline{K}}_{33,\theta\theta} \end{pmatrix} \\
\mathbb{B}_\theta &= \begin{pmatrix} \overline{\overline{\eta}}_{11,\theta\theta} & \overline{\overline{\eta}}_{12,\theta\theta} & \overline{\overline{\eta}}_{13,\theta\theta} \\ \overline{\overline{\eta}}^*_{12,\theta\theta} & \overline{\overline{\eta}}_{22,\theta\theta} & \overline{\overline{\eta}}_{23,\theta\theta} \\ \overline{\overline{\eta}}^*_{13,\theta\theta} & \overline{\overline{\eta}}^*_{23,\theta\theta} & \overline{\overline{\eta}}_{33,\theta\theta} \end{pmatrix}
\end{aligned} \tag{S17}
$$

with $\overline{\overline{K}}_{pp,\theta\theta} = \left(\overline{\omega}_p - i\frac{\gamma_p}{2} + \lambda_\theta \frac{A_p}{4\pi\overline{\omega}_p}\right)$, $\overline{\overline{\lambda}}_{pp',\theta\theta} = \sqrt{\frac{A_p}{2\overline{\omega}_p}}\frac{\lambda_\theta}{2\pi}\sqrt{\frac{A_{p'}}{2\overline{\omega}_{p'}}}$ and $\overline{\overline{\eta}}_{pp',\theta\theta} = \sqrt{\frac{A_p}{2\overline{\omega}_p}}\frac{\lambda_\theta}{2\pi}\left(\sqrt{\frac{A_{p'}}{2\overline{\omega}_{p'}}}\right)^*$. Note that $\mathbb{A}_\theta$ is not hermitian because of the imaginary diagonal terms containing the damping rates, whereas $\mathbb{B}_\theta$ is.

Notably, the form of Eq.S15 bears a strong similarity with the matrix structure of the linear response equation of a quantum system. Indeed, standard linear response theory[4–6] leads to the following general matrix equation

$$
\left[\begin{pmatrix} \mathbb{A} & \mathbb{B} \\ \mathbb{B}^* & \mathbb{A}^* \end{pmatrix} - \omega \begin{pmatrix} \mathbb{I} & 0 \\ 0 & -\mathbb{I} \end{pmatrix}\right] \begin{pmatrix} \mathbb{X} \\ \mathbb{Y} \end{pmatrix} = - \begin{pmatrix} \mathbb{V} \\ \mathbb{V}^* \end{pmatrix} \tag{S18}
$$

where the matrices $\mathbb{A}, \mathbb{B}$ depend on single particle excitations of the system and couplings among them, whereas $\mathbb{X}, \mathbb{Y}$ respectively contain the Fourier transformed resonant and anti-resonant transition amplitudes describing the first-order change in the system density matrix upon perturbation. $\mathbb{V}$ and $\mathbb{V}^*$ respectively store matrix elements of the perturbation.

By comparing Eq.S15 and Eq.S18, it can be observed that the classical PCM-NP equations in the generic dielectric function approach can be exactly mapped to the linear response

equations of a quantum system for each θth BEM eigenmode independently, where

$$
\begin{aligned}
\mathbb{A} &= \mathbb{A}_\theta \\
\mathbb{B} &= \mathbb{B}_\theta \\
\mathbb{X} &= \mathbb{q}_\theta^R \\
\mathbb{Y} &= \mathbb{q}_\theta^A \\
\mathbb{V} &= \mathbb{V}_\theta \, .
\end{aligned}
\tag{S19}
$$

Following this mapping, we can identify the diagonal element $(\mathbb{A}_\theta)_{pp}$ with the pth single particle transition frequency of the quantum NP due to the θth BEM eigenmode ($\overline{\omega}_p + \lambda_\theta \frac{A_p}{4\pi\overline{\omega}_p}$) and corresponding damping rate (γ_p), while $\overline{\overline{\lambda}}_{pp',\theta\theta}$ constitute coupling matrix elements between such transitions. Furthermore, upon introducing the quantized surface charge operator $\hat{q}$ as in Refs..,[2,7] we identify the quantum transition charge sitting on the kth tessera as

$$
\langle 0 | \hat{q}_k | \theta, p \rangle = \left(S^{-1/2} T\right)_{k\theta} \sqrt{\frac{A_p}{2\overline{\omega}_p}\left(1 + \frac{\lambda_\theta}{2\pi}\right)}
\tag{S20}
$$

for each $|\theta, p\rangle$ quantum plasmon mode originating from each pth pole of the generic dielectric function for a given θth BEM eigenmode.

To complete the discussion on the mapping between classical and quantum modes, it is useful to consider the perturbation element $\mathbb{V}_{\theta p}$ and how it can be recast following its definition Eq.S13 and the expression of $\langle 0 | \hat{q}_k | \theta, p \rangle$:

$$
\mathbb{V}_{\theta,p} = \sum_j \mathrm{sgn}(A_p) \left(\sqrt{\frac{A_p}{2\overline{\omega}_p}\left(1 + \frac{\lambda_\theta}{2\pi}\right)} \right)^* \left(T^\dagger S^{-1/2}\right)_{\theta j} V_j = \sum_j \mathrm{sgn}(A_p) \langle \theta, p | \hat{q}_j | 0 \rangle V_j
\tag{S21}
$$

which is the expected form of the perturbation over the tesserae (j index), once the change of sign for negative A_p is accounted for.

On the basis of standard response theory,[4,6] the response matrix can be conveniently recast in its spectral representation. The generalized eigenvalue problem also discussed in

the main text

$$\begin{pmatrix} \mathbb{A}_\theta & \mathbb{B}_\theta \\ \mathbb{B}^*_\theta & \mathbb{A}^*_\theta \end{pmatrix} \boldsymbol{U}_\theta = \begin{pmatrix} \mathbb{I} & 0 \\ 0 & -\mathbb{I} \end{pmatrix} \boldsymbol{U}_\theta \boldsymbol{d}_\theta \tag{S22}$$

specifically reads

$$\left[\begin{pmatrix} \mathbb{A}_\theta & \mathbb{B}_\theta \\ \mathbb{B}^*_\theta & \mathbb{A}^*_\theta \end{pmatrix} - (\omega_{\theta,n} - i\frac{\gamma_{\theta,n}}{2}) \begin{pmatrix} \mathbb{I} & 0 \\ 0 & -\mathbb{I} \end{pmatrix} \right] \boldsymbol{U}_{\theta,n} = 0 \tag{S23}$$

Because of the structure of the response matrix, the eigenvalues come in pairs[6] (if $\omega_{\theta,n} - i\frac{\gamma_{\theta,n}}{2}$ is an eigenvalue, so it is $-\omega_{\theta,n} - i\frac{\gamma_{\theta,n}}{2}$). Thus it is convenient to use an index n running from 1 to N and from -1 to $-N$ to label them and the eigenvectors: $d_{\theta,n} = \omega_{\theta,n} - i\frac{\gamma_{\theta,n}}{2}$ and $d_{\theta,-n} = -\omega_{\theta,n} - i\frac{\gamma_{\theta,n}}{2}$. The $n-th$ eigenvector, i.e. the $n-th$ column of the matrix $\boldsymbol{U}_\theta$, $\boldsymbol{U}_{\theta,n}$, has the structure

$$\boldsymbol{U}_{\theta,n} = \begin{pmatrix} \boldsymbol{X}_{\theta,n} \\ \boldsymbol{Y}_{\theta,n} \end{pmatrix} \quad \boldsymbol{U}_{\theta,-n} = \begin{pmatrix} \boldsymbol{Y}^*_{\theta,n} \\ \boldsymbol{X}^*_{\theta,n} \end{pmatrix} \tag{S24}$$

which leads to

$$\begin{aligned} \langle 0| \hat{q}_k |\theta, n\rangle &= \sum_p \left(\langle 0| \hat{q}_k |\theta, p\rangle X_{\theta,pn} + \langle \theta, p| \hat{q}_k |0\rangle Y_{\theta,pn} \right) = \\ &= \sum_p \left(S^{-1/2}T\right)_{k\theta} \left[\sqrt{\frac{A_p}{2\overline{\omega}_p}\left(1+\frac{\lambda_\theta}{2\pi}\right)} X_{\theta,pn} + \left(\sqrt{\frac{A_p}{2\overline{\omega}_p}\left(1+\frac{\lambda_\theta}{2\pi}\right)} \right)^* Y_{\theta,pn} \right]. \end{aligned} \tag{S25}$$

Since Eq.S22 is a generalized eigenvalue problem, its numerical solution is practically obtained by recasting it into a normal eigenvalue problem where the response matrix features a sign change in the lower row

$$\boldsymbol{U}^{-1}_\theta \begin{pmatrix} \mathbb{I} & 0 \\ 0 & -\mathbb{I} \end{pmatrix} \begin{pmatrix} \mathbb{A}_\theta & \mathbb{B}_\theta \\ \mathbb{B}^*_\theta & \mathbb{A}^*_\theta \end{pmatrix} \boldsymbol{U}_\theta = \boldsymbol{U}^{-1}_\theta \begin{pmatrix} \mathbb{A}_\theta & \mathbb{B}_\theta \\ -\mathbb{B}^*_\theta & -\mathbb{A}^*_\theta \end{pmatrix} \boldsymbol{U}_\theta = \boldsymbol{d}_\theta \tag{S26}$$

which is the actual matrix diagonalization performed in practice. By solving Eq.S15 for $\mathbb{q}_\theta^R = \mathbb{X}_\theta$ and $\mathbb{q}_\theta^A = \mathbb{Y}_\theta$, while making use of Eq.S26 it follows,

$$\begin{pmatrix} \mathbb{X}_\theta \\ \mathbb{Y}_\theta \end{pmatrix} = \boldsymbol{U}_\theta \left[\boldsymbol{d}_\theta - \omega \begin{pmatrix} \mathbb{I} & 0 \\ 0 & \mathbb{I} \end{pmatrix} \right]^{-1} \boldsymbol{U}_\theta^{-1} \begin{pmatrix} \mathbb{I} & 0 \\ 0 & -\mathbb{I} \end{pmatrix} \begin{pmatrix} \mathbb{V}_\theta \\ \mathbb{V}_\theta^* \end{pmatrix} \tag{S27}$$

Note that due to the non-hermicity of $\mathbb{A}_\theta$, $\boldsymbol{U}_\theta^{-1} \neq \boldsymbol{U}_\theta^\dagger$ and so its $n-th$ row cannot, in general, be written in terms of the $\boldsymbol{X}_{\theta,n}$, $\boldsymbol{Y}_{\theta,n}$ vectors, but rather it reads

$$\boldsymbol{U}_{\theta,n}^{-1} = \left(\boldsymbol{Z}_{\theta,n} \ \boldsymbol{W}_{\theta,n} \right) \quad \boldsymbol{U}_{\theta,-n}^{-1} = \left(\boldsymbol{W}_{\theta,n}^* \ \boldsymbol{Z}_{\theta,n}^* \right) \tag{S28}$$

allowing the following identification:

$$\begin{aligned} \langle \theta, n | \hat{q}_k | 0 \rangle &= \sum_p \left(\langle \theta, p | \hat{q}_k | 0 \rangle Z_{\theta,np} - \langle 0 | \hat{q}_k | \theta, p \rangle W_{\theta,np} \right) = \\ &= \sum_p \left(T^\dagger S^{-1/2} \right)_{\theta k} \left[\left(\sqrt{\frac{A_p}{2\varpi_p} \left(1 + \frac{\lambda_\theta}{2\pi} \right)} \right)^* Z_{\theta,np} - \sqrt{\frac{A_p}{2\varpi_p} \left(1 + \frac{\lambda_\theta}{2\pi} \right)} W_{\theta,np} \right]. \end{aligned} \tag{S29}$$

Note that in this most general case, rigorously speaking $\langle 0 | \hat{q}_k | \theta, n \rangle^* \neq \langle \theta, n | \hat{q}_k | 0 \rangle$ (although in practice it holds $\langle 0 | \hat{q}_k | \theta, n \rangle^* \approx \langle \theta, n | \hat{q}_k | 0 \rangle$); thus the notation should be understood to refer only approximately to a set of excited states $|\theta, n\rangle$.

On the basis of eqs.S22-S29 and response theory,[6] the spectral representation of the dipole-dipole polarizability of the quantum NP becomes

$$\begin{aligned} \alpha_{ab}(\omega) = \sum_{\theta,n} & \frac{\langle 0 | \hat{\mu}_a | \theta, n \rangle \sum_p \mathrm{sgn}(A_p) (\langle \theta, p | \hat{\mu}_b | 0 \rangle Z_{\theta,np} - \langle 0 | \hat{\mu}_b | \theta, p \rangle W_{\theta,np})}{\omega_{\theta,n} - \omega - i \frac{\gamma_{\theta,n}}{2}} + \\ & \frac{\langle 0 | \hat{\mu}_a | \theta, n \rangle^* \left(\sum_p \mathrm{sgn}(A_p) (\langle \theta, p | \hat{\mu}_b | 0 \rangle Z_{\theta,np} - \langle 0 | \hat{\mu}_b | \theta, p \rangle W_{\theta,np}) \right)^*}{\omega_{\theta,n} + \omega + i \frac{\gamma_{\theta,n}}{2}} \end{aligned} \tag{S30}$$

When there are no negative A_p, this equation can be further simplify to:

$$\alpha_{ab}(\omega) = \sum_{\theta,n} \frac{\langle 0| \hat{\mu}_a |\theta, n\rangle \langle \theta, n| \hat{\mu}_b |0\rangle}{\omega_{\theta,n} - \omega - i\frac{\gamma_{\theta,n}}{2}} + \frac{\langle 0| \hat{\mu}_a |\theta, n\rangle^* \langle \theta, n| \hat{\mu}_b |0\rangle^*}{\omega_{\theta,n} + \omega + i\frac{\gamma_{\theta,n}}{2}} \tag{S31}$$

where $\hat{\mu}_a = \hat{q}_k \vec{r}_{k,a}$ with $\vec{r}_{k,a}$ being the ath component of the position vector pointing to the kth surface NP tessera.

2 Plasmon-molecule Hamiltonian in the generic dielectric function framework

The Q-PCM-NP quantum model described in SI Section 1 can be coupled to a quantum chemistry molecular description to investigate plexcitonic effects at full-quantum level.

Following Ref. 8 and considering the results of SI 1, the full plasmon-molecule Hamiltonian in the generic dielectric function Q-PCM-NP approach can be written as

$$\hat{H}_{\text{tot}} = \hat{H}_{\text{mol}} + \sum_{\theta,n} \left(\omega_{\theta,n} - i\frac{\gamma_{\theta,n}}{2}\right) \hat{b}^{\dagger}_{\theta,n}\hat{b}_{\theta,n} + \sum_{\theta,n,k} \hat{V}_k \left(\langle \theta, n| \hat{q}_k |0\rangle \hat{b}^{\dagger}_{\theta,n} + \langle 0| \hat{q}_k |\theta, n\rangle \hat{b}_{\theta,n}\right) \tag{S32}$$

where $\hat{H}_{\text{mol}}$ is the standard molecular electronic Hamiltonian and $\hat{V}_k$ is the molecular electrostatic potential operator evaluated at centroid of the k-th surface tessera of the discretized NP.

Solving the full Hamiltonian in Eq.S32 at ab initio level is often computationally demanding due to the additional plasmonic degrees of freedom beyond the already costly electronic structure treatment. A viable strategy to reduce this cost is to first calculate a given number of electronic states of the isolated molecule and subsequently couple them to the plasmonic modes, as in Ref. 9. This alternative is, in principle, exact in the limit where all plasmonic modes of the NP are included, thereby recovering the full configuration-interaction limit. In practice one has to first solve the molecular Hamiltonian $\hat{H}_{\text{mol}}$ and subsequently couple its

eigenstates to the plasmonic system. Following this line, upon denoting the eigenstates of $\hat{H}_{\text{mol}}$ as $|g\rangle$ for the molecular ground state and $|e\rangle$ for one generic excited state, $\hat{H}_{\text{tot}}$ can be expressed as,

$$
\begin{aligned}
\hat{H}_{\text{tot}} = \quad & \omega_g |g\rangle \langle g| + \sum_e \left(\omega_e - i\frac{\gamma_e}{2}\right) |e\rangle \langle e| + \sum_{\theta,n} \left(\omega_{\theta,n} - i\frac{\gamma_{\theta,n}}{2}\right) \hat{b}^{\dagger}_{\theta,n}\hat{b}_{\theta,n} + \\
& + \sum_{\theta,n,k} \hat{V}_k \left(\langle \theta, n| \hat{q}_k |0\rangle \hat{b}^{\dagger}_{\theta,n} + \langle 0| \hat{q}_k |\theta, n\rangle \hat{b}_{\theta,n} \right)
\end{aligned}
\tag{S33}
$$

where ω_g and ω_e are the molecular energies of ground and excited states, respectively, while γ_e is a phenomenological damping rate to account for the finite lifetime of the molecular excited state $|e\rangle$.

Upon introducing the molecular identity operator $\mathbb{1}_{\text{mol}} = |g\rangle \langle g| + \sum_e |e\rangle \langle e|$ before and after $\hat{V}_k$ in Eq.S33, it follows

$$
\begin{aligned}
\hat{H}_{\text{tot}} = \quad & \omega_g |g\rangle \langle g| + \sum_e \left(\omega_e - i\frac{\gamma_e}{2}\right) |e\rangle \langle e| + \sum_{\theta,n} \left(\omega_{\theta,n} - i\frac{\gamma_{\theta,n}}{2}\right) \hat{b}^{\dagger}_{\theta,n}\hat{b}_{\theta,n} + \\
& + \sum_{\theta,n,k} \left(\hat{V}_k^{gg} |g\rangle \langle g| + \sum_e \hat{V}_k^{ee} |e\rangle \langle e| \right) \left(\langle \theta, n| \hat{q}_k |0\rangle \hat{b}^{\dagger}_{\theta,n} + \langle 0| \hat{q}_k |\theta, n\rangle \hat{b}_{\theta,n} \right) \\
& + \sum_{\theta,n,k,e} \left(\hat{V}_k^{eg} |e\rangle \langle g| + \hat{V}_k^{ge} |g\rangle \langle e| \right) \left(\langle \theta, n| \hat{q}_k |0\rangle \hat{b}^{\dagger}_{\theta,n} + \langle 0| \hat{q}_k |\theta, n\rangle \hat{b}_{\theta,n} \right) \\
& + \sum_{\theta,n,k,e\neq e'} \hat{V}_k^{ee'} |e\rangle \langle e'| \left(\langle \theta, n| \hat{q}_k |0\rangle \hat{b}^{\dagger}_{\theta,n} + \langle 0| \hat{q}_k |\theta, n\rangle \hat{b}_{\theta,n} \right)
\end{aligned}
\tag{S34}
$$

where $\hat{V}_k^{ge}$ is a shorthand notation for $\langle g| \hat{V}_k |e\rangle$. Note that the diagonal terms of the plasmon-molecule interaction (second line of Eq.S34) are NP polarization terms that lead to a correction of the bare molecular excitation energies $\omega_e - \omega_g$. The last line of the equation contains terms that promote a mixing of the bare molecular excited states due to molecule-NP polarization effects.

Eq.S34 can be further simplified upon applying the usual rotating-wave approximation

to the interaction terms in the last two lines of Eq.S34, leading to

$$\begin{aligned}
\hat{H}_{\mathrm{tot}} = \quad & \omega_g \left|g\right\rangle \left\langle g\right| + \sum_e \left(\omega_e - i\frac{\gamma_e}{2}\right) \left|e\right\rangle \left\langle e\right| + \sum_{\theta,n} \left(\omega_{\theta,n} - i\frac{\gamma_{\theta,n}}{2}\right) \hat{b}^\dagger_{\theta,n}\hat{b}_{\theta,n} + \\
& + \sum_{\theta,n,k} \left(\hat{V}^{gg}_k \left|g\right\rangle \left\langle g\right| + \sum_e \hat{V}^{ee}_k \left|e\right\rangle \left\langle e\right|\right) \left(\left\langle \theta,n\right| \hat{q}_k \left|0\right\rangle \hat{b}^\dagger_{\theta,n} + \left\langle 0\right| \hat{q}_k \left|\theta,n\right\rangle \hat{b}_{\theta,n}\right) \\
& + \sum_{\theta,n,k,e} \hat{V}^{eg}_k \left(\left\langle \theta,n\right| \hat{q}_k \left|0\right\rangle \hat{b}^\dagger_{\theta,n} \left|g\right\rangle \left\langle e\right| + \left\langle 0\right| \hat{q}_k \left|\theta,n\right\rangle \hat{b}_{\theta,n} \left|e\right\rangle \left\langle g\right|\right) \\
& + \sum_{\theta,n,k,e<e'} \hat{V}^{ee'}_k \left(\left\langle \theta,n\right| \hat{q}_k \left|0\right\rangle \hat{b}^\dagger_{\theta,n} \left|e\right\rangle \left\langle e'\right| + \left\langle 0\right| \hat{q}_k \left|\theta,n\right\rangle \hat{b}_{\theta,n} \left|e'\right\rangle \left\langle e\right|\right) . \qquad \text{(S35)}
\end{aligned}$$

Note that the molecular transition potential terms are often real in standard quantum chemistry codes and so it holds $\hat{V}^{eg}_k = \hat{V}^{ge}_k$ and $\hat{V}^{ee'}_k = \hat{V}^{e'e}_k$, which is considered in Eq.S35.

In the present work the Hamiltonian of Eq.S35 is considered and diagonalized in the single-excitation subspace $(\left|g\right\rangle\otimes\left|0_{\theta,n}\right\rangle, \left|e\right\rangle\otimes\left|0_{\theta,n}\right\rangle, \left|g\right\rangle\otimes\left|1_{\theta,n}\right\rangle)$ to investigate plasmon-molecule coupling effects. The use of the full Hamiltonian of Eq.S34 extending the basis states up to the second-excitation manifold leads to small negligible differences for the system investigated in the present work (see also main text, Fig.3), as it is shown in Fig.S5.

The Hamiltonian of eq.S35 is the same Hamiltonian discussed in main text (Eq.30).

3 Computational details

Simulations reported in main text have been performed on a test case NP of ellipsoidal shape, whose main axis is 10 nm long and the short axis is 6 nm long (Fig. 1). The surface mesh has been created with the Gmsh[10] code and features 1371 surface tesserae. The Ag Brendel-Bormann[11] and Au Etchegoin[12] frequency-dependent data of the metal dielectric functions were fitted as a sum of 4 and 6 DL poles,[1] respectively. The fitting $f(\omega)$ functions are shown in Figs. S1-S2 and the corresponding fitting parameters are given in Tables S1-S2. As mentioned in main text, only the BEM dipolar eigenmode oriented along the main axis of the NP (x axis) $\theta = \theta_{\mathrm{dip}}$ has been considered for the results presented in Fig.2, since it

is the only bright one contributing to $\alpha_{xx}(\omega)$ in the quasi-static limit, which is the quantity discussed in Fig.2. The same approximation has been coherently applied to the classical PCM-NP equations by setting to zero the contribution to the classical response charges from all other modes.

When coupling with the quinolone molecule is investigated, the same ellipsoidal NP shape has been used but the number of tesserae in the surface region closer to the molecule was increased to improve numerically the evaluation of the the molecule-NP electrostatic coupling terms. In that case 2122 tesserae were used. The quinolone molecule of Fig.3 (main text) was described at CIS/6-31g** level of theory using a locally modified version of the GAMESS code[13] to obtain the molecular transition potential matrix elements evaluated at the NP surface.

The DL dielectric function that is used for the results of main text Fig.4 is of the form,[2]

$$\epsilon_{\mathrm{DL}}(\omega) = 1 + \frac{\Omega_{\mathrm{DL}}^2}{\omega_{\mathrm{DL}}^2 - \omega^2 - i\gamma_{\mathrm{DL}}\omega} \tag{S36}$$

and the following parameters $\Omega_{\mathrm{DL}} = 4.78\,\mathrm{eV}\,;\omega_{\mathrm{DL}} = 2.17\,\mathrm{eV}\,;\gamma_{\mathrm{DL}} = 0.219\,\mathrm{eV}$ where used to obtain the Q-PCM-NP DL modes according to the methodology described in Ref. 2.

The molecular damping rate considered for the results in main text is $\gamma_e = 6.6\,\mathrm{meV}$ (decay time $\approx$ 100fs).

For all results of Fig.3 (main text) about the molecule-NP coupling, all plasmonic modes arising from the first 45 geometric BEM eigenmodes (SI 1) have been considered for solving $\hat{H}_{\mathrm{tot}}$ (Eq.S35). In the case of the DL model this leads to 45 plasmonic modes, whereas for the generic dielectric function this leads to $4 \times 45 = 180$ plasmonic modes due to the 4 poles used to fit the Ag dielectric function data. Convergence of the results of Fig.3 (main text) was observed with such number of modes.

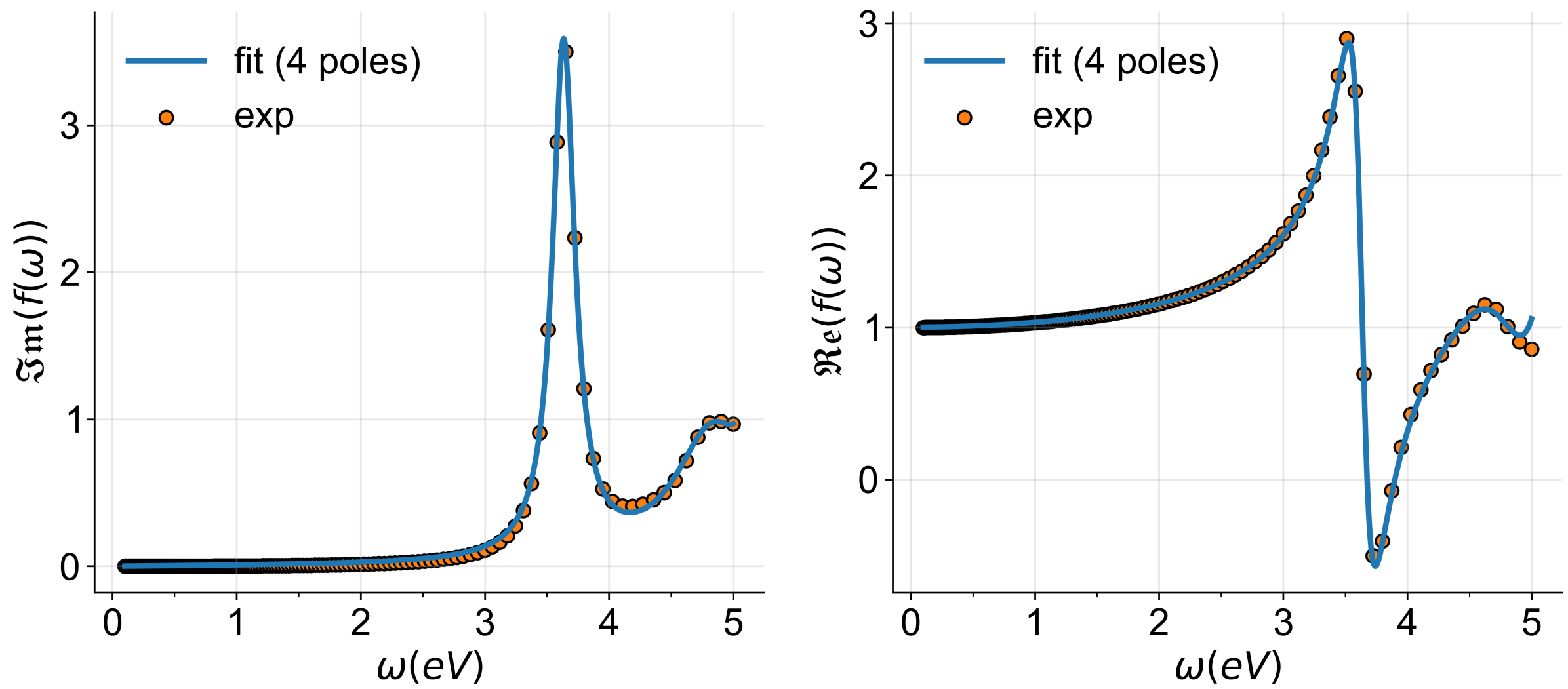

Figure S1: Fitting of Ag $f(\omega)$ function with 4 poles to Brendel-Bormann[11] reference data (orange dots). The fitting result is the solid blue line. Left and right panels respectively show imaginary and real parts of $f(\omega)$. The fitting parameters are given in Table S1.

Table S1: Fitting pole parameters of $f(\omega)$ for Ag Brendel-Bormann[11] reference data.

pole n.	ω_p (eV)	γ_p (eV)	A_p (eV^2)
1	3.64	0.219	2.79
2	4.85	0.763	3.33
3	5.18	0.0861	1.51
4	9.89	0.0	58.1

Table S2: Fitting pole parameters of $f(\omega)$ for Au Etchegoin[12] reference data.

pole n.	ω_p (eV)	γ_p (eV)	A_p (eV^2)
1	2.51	0.573	0.674
2	2.59	1.76	-5.19
3	2.85	2.34	7.90
4	5.24	9.06	15.4
5	8.39	7.52	63.1
6	42.4	0	643

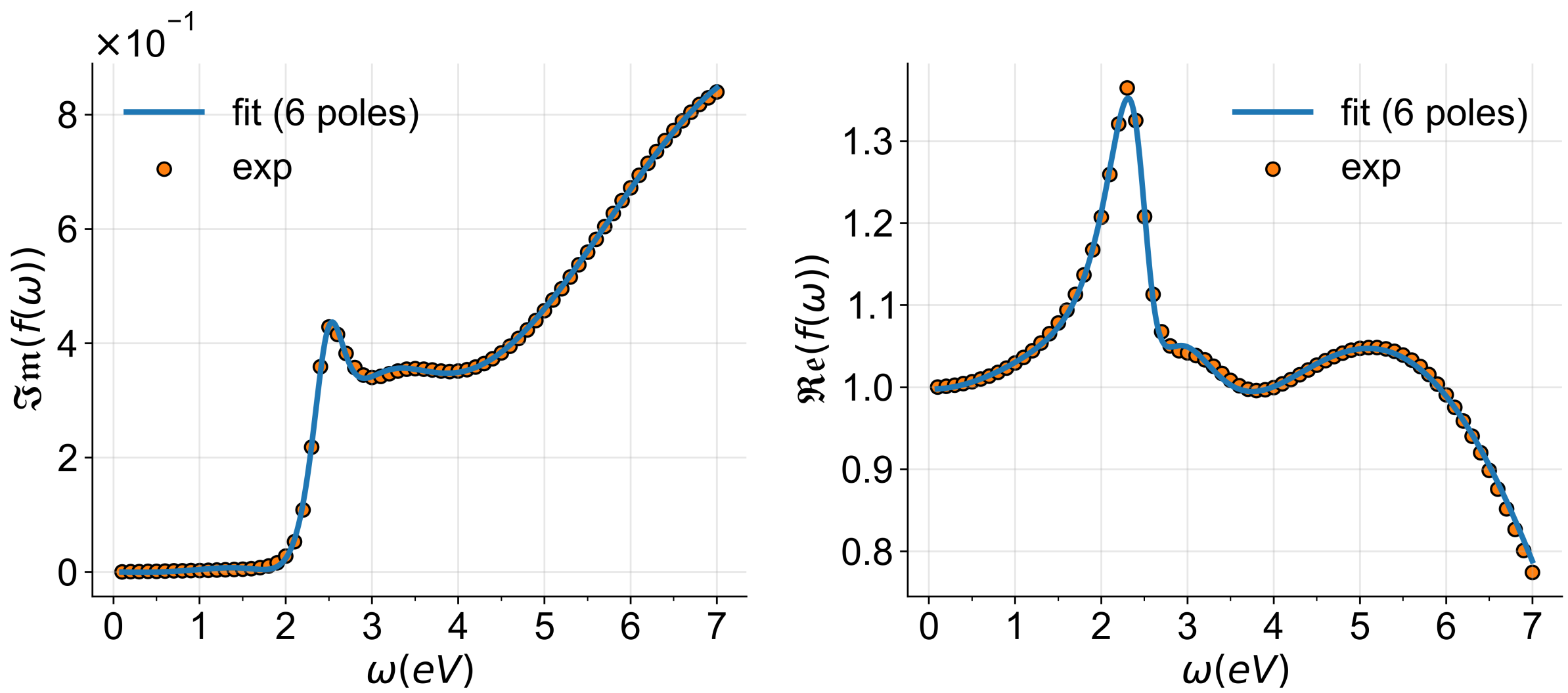

Figure S2: Fitting of Au $f(\omega)$ function with 6 poles to Etchegoin[12] reference data (orange dots). The fitting result is the solid blue line. Left and right panels respectively show imaginary and real parts of $f(\omega)$. The fitting parameters are given in Table S2

4 Real part of $\alpha_{xx}(\omega)$ for the ellipsoidal NP of Figs.1-2

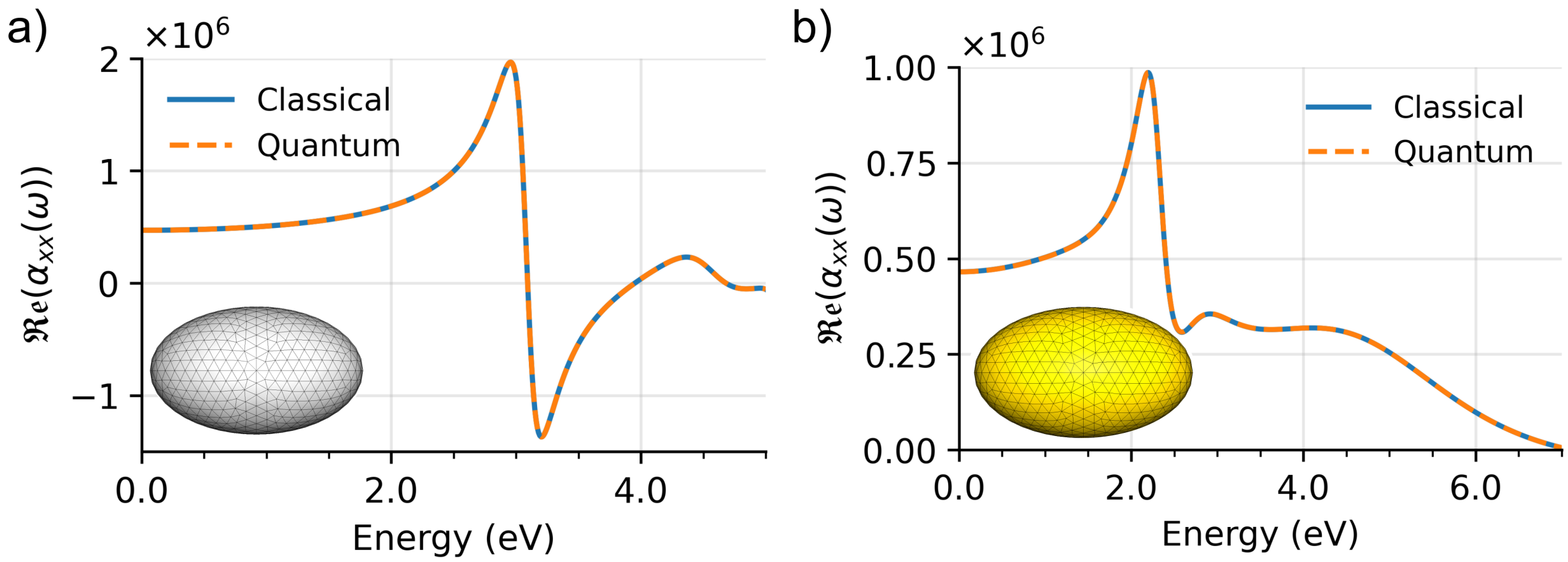

Figure S3: Real part of the xx component of the dipole-dipole polarizability tensor for the ellipsoidal NP (Fig. 1) when the $f(\omega)$ function is fitted to Ag Brendel-Bormann[11] (a) or Au Etchegoin[12] (b) reference data. The classical PCM-NP (solid blue) and quantum Q-PCM-NP results (dashed orange) are shown.

5 Results for a gold spherical NP

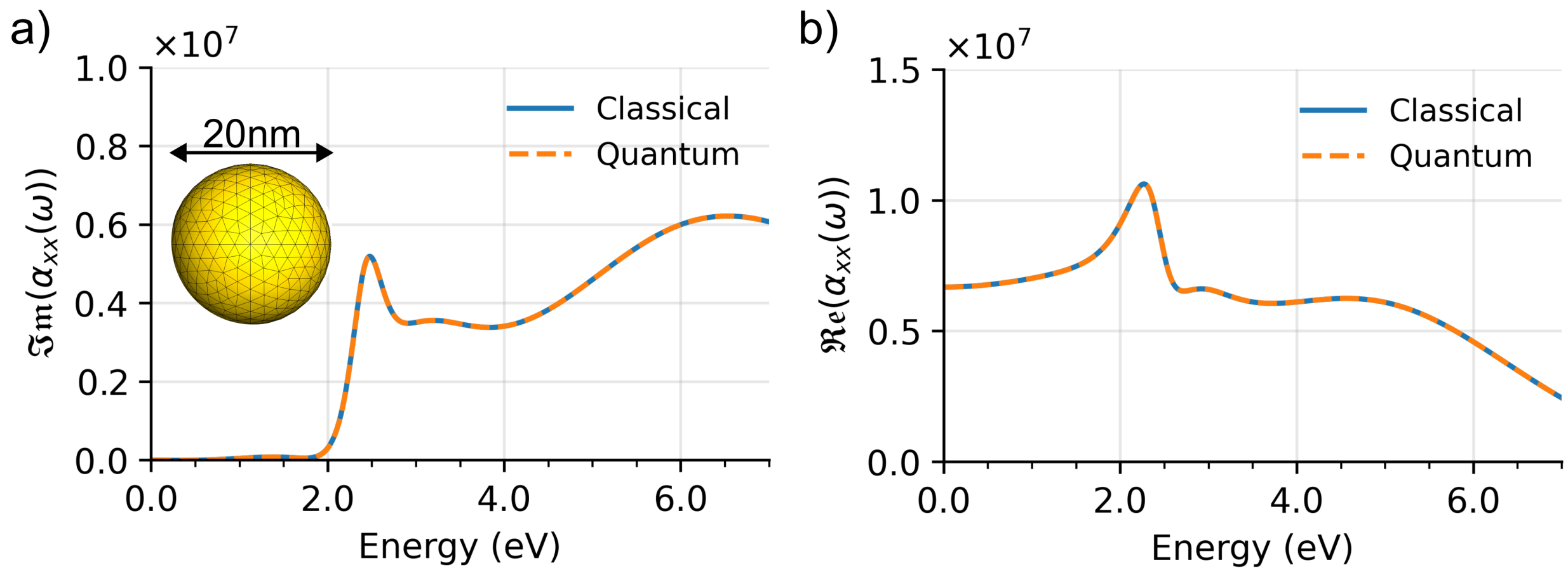

Figure S4: 1) Imaginary and real part 2) of the xx component of the dipole-dipole polarizability tensor for a gold spherical NP featuring a diameter of 20 nm NP. The same $f(\omega)$ function as in the case of the ellipsoidal Au NP is used. The classical PCM-NP (solid blue) and quantum Q-PCM-NP results (dashed orange) are shown. Despite spherical NPs have three degenerate dipolar modes only one dipolar mode (oriented along x) is considered here for simplicity.

6 Impact of the rotating wave approximation and the second excitation manifold

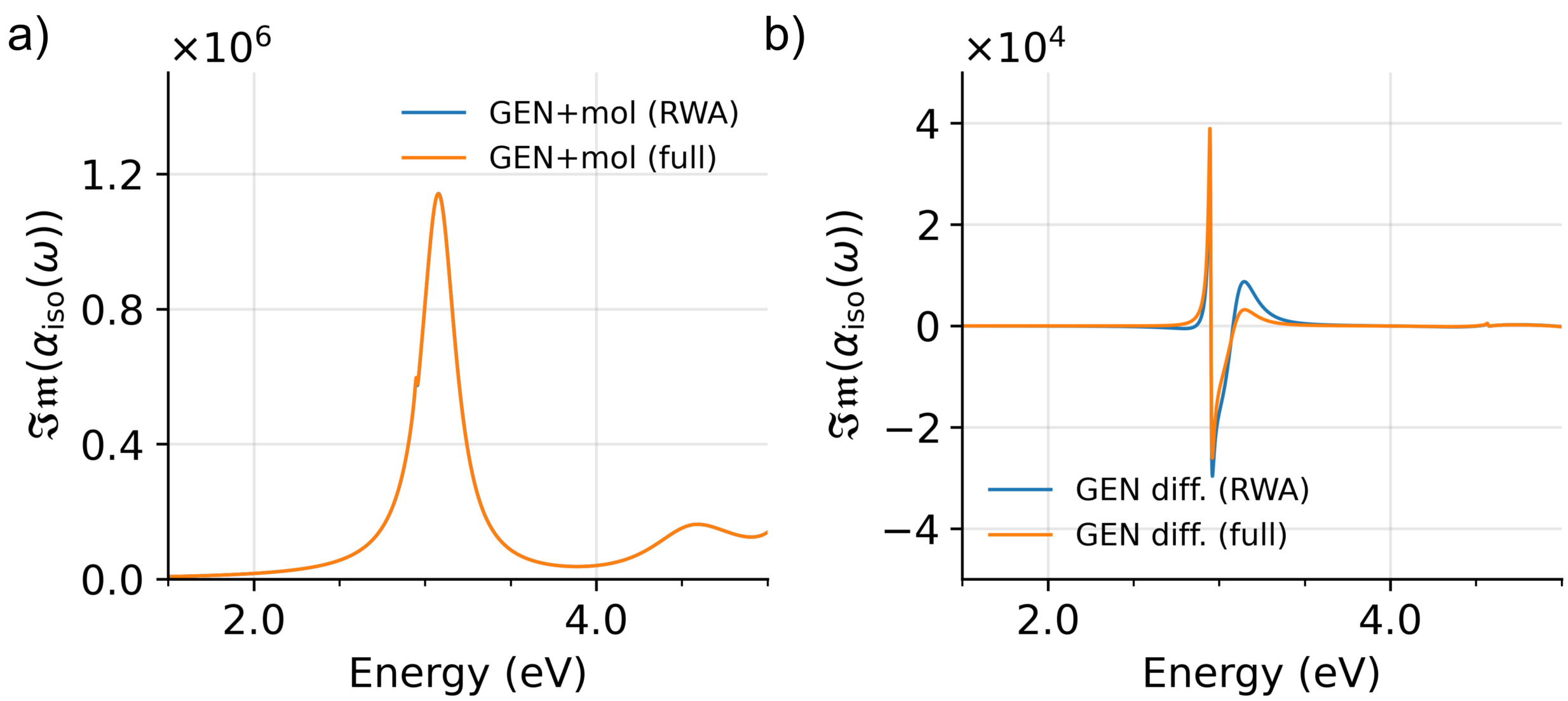

Figure S5: a) Imaginary part of the isotropic polarizability of the coupled nanoparticle–molecule (NP–molecule) system obtained using the generic dielectric-function quantum modes. The molecule–NP coupling is described by diagonalizing either Eq. S35 (blue) or the full Hamiltonian of Eq. S34 (orange) and only the BEM dipolar mode oriented along the NP-molecule axis is considered here for simplicity. In the case of the full Hamiltonian Eq. S34 (orange) the basis states of the second-excitation manifold are also included. b) Corresponding difference between the isotropic polarizability of the coupled system shown in panel (a) and the isotropic polarizability of the bare plasmonic nanoparticle.

References

(1) Dall'Osto, G.; Gil, G.; Pipolo, S.; Corni, S. Real-time dynamics of plasmonic resonances in nanoparticles described by a boundary element method with generic dielectric function. *J. Chem. Phys.* **2020**, *153*.

(2) Fregoni, J.; Granucci, G.; Persico, M.; Corni, S. Strong coupling with light enhances the photoisomerization quantum yield of azobenzene. *Chem* **2020**, *6*, 250–265.

(3) Corni, S.; Pipolo, S.; Cammi, R. Equation of motion for the solvent polarization apparent charges in the polarizable continuum model: Application to real-time TDDFT. *J. Phys. Chem. A* **2015**, *119*, 5405–5416.

(4) Norman, P.; Ruud, K.; Saue, T. *Principles and practices of molecular properties: Theory, modeling, and simulations*; Wiley, Hoboke, New Jersey, U.S., 2018.

(5) Ye, H.; Becca, J. C.; Jensen, L. Modeling the near-field effect on molecular excited states using the discrete interaction model/quantum mechanical method. *J. Chem. Phys.* **2024**, *160*.

(6) McWeeny, R. *Methods of Molecular Quantum Mechanics*, 2nd ed.; Academic Press, San Diego, California, U.S., 1992.

(7) Guido, C. A.; Rosa, M.; Cammi, R.; Corni, S. An open quantum system theory for polarizable continuum models. *J. Chem. Phys.* **2020**, *152*.

(8) Fregoni, J.; Haugland, T. S.; Pipolo, S.; Giovannini, T.; Koch, H.; Corni, S. Strong coupling between localized surface plasmons and molecules by coupled cluster theory. *Nano Lett.* **2021**, *21*, 6664–6670.

(9) Romanelli, M.; Corni, S. Identifying Differences between Semiclassical and Full-Quantum Descriptions of Plexcitons. *J. Phys. Chem. Lett.* **2024**, *15*, 9326–9334.

(10) Geuzaine, C.; Remacle, J.-F. Gmsh: A 3-D finite element mesh generator with built-in pre-and post-processing facilities. *Int. J. Numer. Methods Eng.* **2009**, *79*, 1309–1331.

(11) Rakić, A. D.; Djurišić, A. B.; Elazar, J. M.; Majewski, M. L. Optical properties of metallic films for vertical-cavity optoelectronic devices. *Appl. Opt.* **1998**, *37*, 5271–5283.

(12) Etchegoin, P. G.; Le Ru, E.; Meyer, M. An analytic model for the optical properties of gold. *J. Chem. Phys.* **2006**, *125*.

(13) Schmidt, M. W.; Baldridge, K. K.; Boatz, J. A.; Elbert, S. T.; Gordon, M. S.; Jensen, J. H.; Koseki, S.; Matsunaga, N.; Nguyen, K. A.; Su, S.; others General atomic and molecular electronic structure system. *J. Comput. Chem.* **1993**, *14*, 1347–1363.
